# Human Papillomavirus Type 8 Interferes with a Novel C/EBPβ-Mediated Mechanism of Keratinocyte CCL20 Chemokine Expression and Langerhans Cell Migration

**DOI:** 10.1371/journal.ppat.1002833

**Published:** 2012-07-26

**Authors:** Tanya Sperling, Monika Ołdak, Barbara Walch-Rückheim, Claudia Wickenhauser, John Doorbar, Herbert Pfister, Magdalena Malejczyk, Sławomir Majewski, Andrew C. Keates, Sigrun Smola

**Affiliations:** 1 Institute of Virology and Center for Molecular Medicine Cologne, University of Cologne, Cologne, Germany; 2 Institute of Virology, Saarland University, Homburg/Saar, Germany; 3 Department of Histology and Embryology Center of Biostructure Research, Medical University of Warsaw, Warsaw, Poland; 4 Institute of Pathology, University of Cologne, Cologne, Germany; 5 Institute of Pathology, University of Leipzig, Leipzig, Germany; 6 Division of Virology, National Institute for Medical Research, Mill Hill, London, United Kingdom; 7 Department of Dermatology and Venereology, Medical University of Warsaw, Warsaw, Poland; 8 Division of Gastroenterology, Beth Israel Deaconess Medical Center, Boston, Massachusetts, United States of America; Fred Hutchinson Cancer Research Center, United States of America

## Abstract

Infection with genus beta human papillomaviruses (HPV) is implicated in the development of non-melanoma skin cancer. This was first evidenced for HPV5 and 8 in patients with epidermodysplasia verruciformis (EV), a genetic skin disease. So far, it has been unknown how these viruses overcome cutaneous immune control allowing their persistence in lesional epidermis of these patients. Here we demonstrate that Langerhans cells, essential for skin immunosurveillance, are strongly reduced in HPV8-positive lesional epidermis from EV patients. Interestingly, the same lesions were largely devoid of the important Langerhans cells chemoattractant protein CCL20. Applying bioinformatic tools, chromatin immunoprecipitation assays and functional studies we identified the differentiation-associated transcription factor CCAAT/enhancer binding protein β (C/EBPβ) as a critical regulator of *CCL20* gene expression in normal human keratinocytes. The physiological relevance of this finding is supported by our in vivo studies showing that the expression patterns of CCL20 and nuclear C/EBPβ converge spatially in the most differentiated layers of human epidermis. Our analyses further identified C/EBPβ as a novel target of the HPV8 E7 oncoprotein, which co-localizes with C/EBPβ in the nucleus, co-precipitates with it and interferes with its binding to the CCL20 promoter in vivo. As a consequence, the HPV8 E7 but not E6 oncoprotein suppressed C/EBPβ-inducible and constitutive *CCL20* gene expression as well as Langerhans cell migration. In conclusion, our study unraveled a novel molecular mechanism central to cutaneous host defense. Interference of the HPV8 E7 oncoprotein with this regulatory pathway allows the virus to disrupt the immune barrier, a major prerequisite for its epithelial persistence and procarcinogenic activity.

## Author Summary

Human papillomaviruses (HPVs) infect squamous epithelial cells of skin or mucosa, giving rise to hyperproliferative lesions. A subgroup of “high-risk” genus alpha HPVs is associated with human anogenital malignancies, e.g. cervical cancer. The skin carcinogenic potential of genus beta HPV types, such as HPV8, is fully accepted in epidermodysplasia verruciformis (EV) patients and their contribution to the development of non-melanoma skin cancer in the general population is under investigation. This genetic disorder serves as a model disease for studying the immunobiology, viral persistence and molecular mechanisms of HPV-induced skin carcinogenesis. Here, we demonstrate that antigen-presenting Langerhans cells and the Langerhans cell attracting chemokine CCL20 are strongly reduced in lesional skin of EV patients. We show that the HPV8 encoded E7 oncoprotein substantially contributes to this disturbance of cutaneous innate immune control. Our data define a novel mechanism of C/EBPβ-dependent *CCL20* gene regulation. HPV8 E7 directly interacts with this transcription factor, interferes with its binding to the CCL20 promoter and suppresses keratinocyte CCL20 expression as well as Langerhans cell migration. Our study unravels a molecular mechanism of virus-host interaction critical for evading host immune defense and providing a microenvironment that is conducive to persistent HPV infection and skin carcinogenesis.

## Introduction

Human papillomaviruses (HPVs) are double-stranded DNA viruses, which infect epithelial cells of skin or mucosa and subsequently induce hyperproliferative lesions. HPVs are classified into five genera based on phylogeny, genome organization, biology and pathogenicity [1]. Malignant progression of genus beta HPV-induced lesions, in particular in the case of HPV5 or 8, was first observed in patients suffering from epidermodysplasia verruciformis (EV), an inherited skin disease [2], [3]. The oncogenic potential of genus beta HPVs has been clearly demonstrated in vitro and in transgenic mouse models recently [4]–[7]. A putative contribution of beta HPV types to skin carcinogenesis is also under investigation in immunocompromised individuals and in the general population [8]–[10].

EV begins early in life with the development of disseminated flat warts or scaly plaques in genetically predisposed individuals infected with beta HPV types. In more than half of these patients benign skin lesions gradually progress to precancerous lesions and invasive non-melanoma skin cancer, particularly at sun-exposed areas of the skin [11]. In EV patients the viruses persist during the process of carcinogenesis. Thus, premalignant lesions and carcinomas harbor the viruses throughout the lesions and express high levels of the viral oncoproteins [12], [13]. The genetic defect in the majority of EV patients has been identified as inactivating mutation in either EVER1 or EVER2 [14]. These genes code for endoplasmic reticulum channel proteins regulating cellular zinc balance. It has been speculated that mutated EVER proteins may enhance beta HPV gene expression [15]. However, it is still unresolved how the viruses escape immune control and manage to persist in the skin of EV patients.

Induction of immune responses critically depends on the activity of antigen-presenting cells, such as Langerhans cells (LC) in the skin. These cells reside in the epidermis and in response to danger signals, such as UV irradiation, leave the skin, migrate to local lymph nodes and initiate activation of effector cells [16]. Thus, Langerhans cells are key regulators of immune responses in skin bridging innate and adaptive immunity. Lesional areas of EV epidermis were found to be largely devoid of cells expressing MHC class II (HLA-DR) and CD1a, a marker of Langerhans cells [17]–[19]. It was hypothesized that this might contribute to HPV persistence in EV lesions.

The CC chemokine ligand 20 (CCL20) also known as macrophage inflammatory protein-3α, plays a decisive role in the recruitment of Langerhans cell precursors into the epidermis [20]–[22]. CCL20 is expressed constitutively and is inducible in human skin [20], [23], [24]. Also in vitro CCL20 is constitutively expressed at low levels by cultured human keratinocytes and is potently up-regulated upon stimulation with proinflammatory mediators [25]–[27] as well as phorbol 12-myristate 13-acetate (PMA) [28]. Tumor necrosis factor-α-(TNF-α)- and poly(I∶C)-induced expression of CCL20 mainly depends on the activity of the inflammatory transcription factor NF-κB (nuclear-factor kappa B) [29]–[31]. Whether or not CCL20 expression is altered in EV lesions has not been investigated so far.

Here we show that CCL20 is expressed in the uppermost differentiated layers of normal human epidermis but is almost absent in HPV8-positive lesional skin of EV patients. To elucidate the underlying molecular mechanisms, we investigated the transcriptional regulation of CCL20. In this study, we identified CCAAT/enhancer-binding protein β (C/EBPβ), a cellular differentiation-associated transcription factor, as a strong activator of CCL20 transcription in human keratinocytes. Our data demonstrate for the first time that C/EBPβ-mediated up-regulation of CCL20 is directly targeted by the HPV8 E7 protein. This leads to the impairment of Langerhans cell migration.

## Results

### Langerin-positive cells and CCL20 expression are strongly reduced in HPV8-positive skin lesions of EV patients

**Figure 1:**
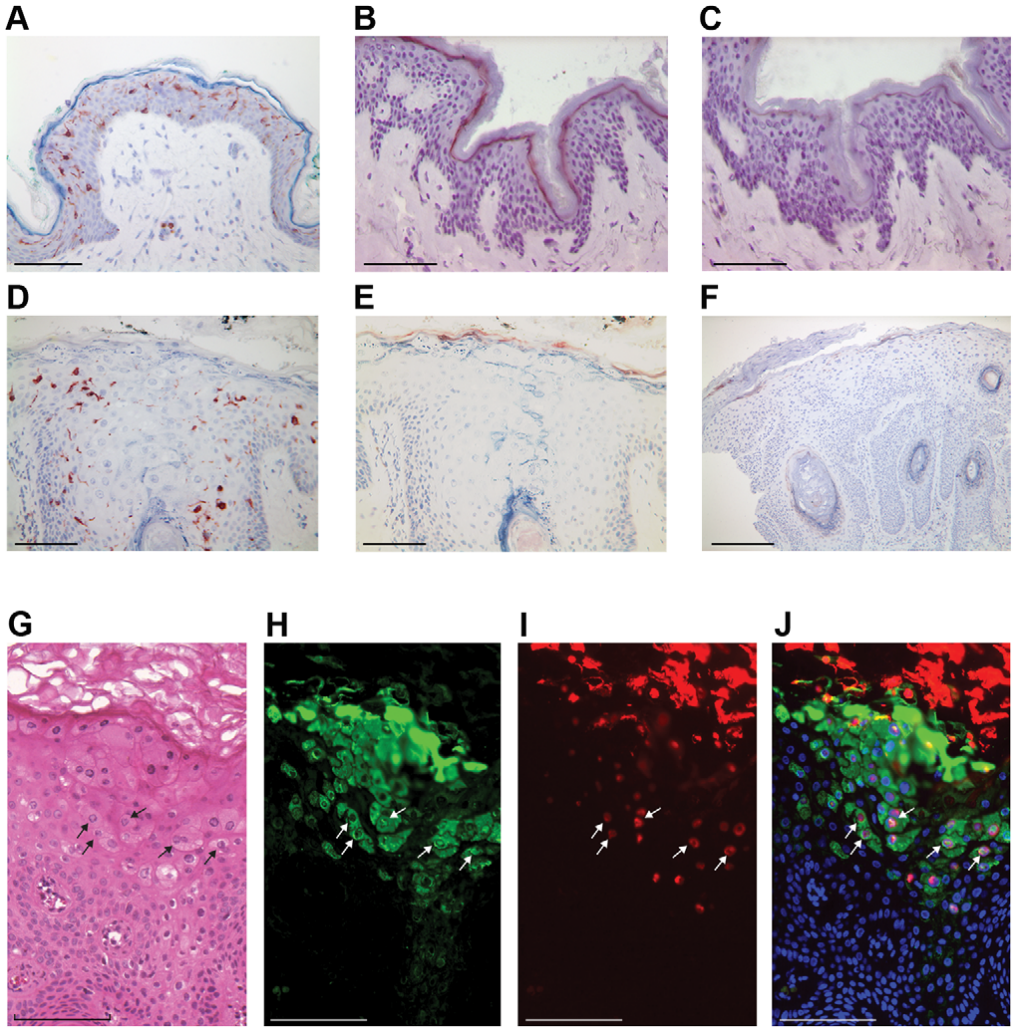
Langerin-positive cells and CCL20 expression are reduced in lesional skin of EV patients. Sections of normal human skin (A, B, C) and lesional skin from EV patients (D–J) were stained using antibodies against langerin (A, D) or CCL20 (B, C, E, F) and hematoxylin. To verify the specificity of the immunohistochemical reaction, recombinant CCL20 protein was used in a blocking experiment (C). Lesion positive for HPV8 only (D, E, G–J). Lesion coinfected with HPV5 and HPV8 (F). Validation of active infection was visualized by hematoxylin eosin staining to reveal the characteristic E4 cytoplasmic inclusions typically found in EV lesions (G) along with immunofluorescence staining for the HPV8 E4 protein (green (H)) and FISH staining to identify the presence of amplified HPV genomes (red (I)). The merged image showing the onset of genome amplification in E4-positive cells is shown in J. Cell nuclei are visualized in J by counterstaining with DAPI. Selected cells double positive for E4 protein and HPV8 DNA are marked with arrows. Bars correspond to 100 µm in A–E and G–J, and to 200 µm in F.

We compared normal human skin with HPV8-positive skin biopsies derived from EV patients. Immunohistochemical analysis demonstrated that langerin-expressing cells were evenly distributed throughout the normal epidermis (Figure 1A). In contrast, lesions from the skin of EV patients with productive HPV8 infection (Figure 1G–J) harbored significantly reduced numbers of langerin-positive cells (Figure 1D, Figure S1A). These lesions were associated with characteristic cytoplasmic inclusions similar to those previously reported for gamma and mu HPV types [32], [33]. As expected, these cells stained strongly with antibodies to the HPV8 E4 protein (green staining in Figure 1H and J) [34], and the same cells also showed nuclear FISH signals for HPV8 DNA (red signal in Figure 1I and J) proving the presence of an active genus beta HPV infection throughout the lesion. As with other HPV types, the onset of HPV8 genome amplification was found to coincide closely with E4 expression in the upper epithelial layers. The viral particles are assembled in the nucleus and as nuclear breakdown occurs the virus is released into the cytoplasm of the most differentiated cells in the stratum corneum. Our data confirmed previous studies showing a strong reduction of MHC class II- and CD1a-positive cells in productively infected EV skin lesions [18].

In a second set of experiments, we investigated expression of the Langerhans cell chemoattractant protein CCL20 in the biopsies. CCL20 was detected in normal human epidermis as a uniformly stained band in the uppermost differentiated layers of the stratum granulosum (Figure 1B). The staining showed a cell-associated cytoplasmic distribution pattern of CCL20 at a higher magnification (Figure S1B). Specificity of CCL20 staining was confirmed in a blocking experiment using recombinant CCL20 (Figure 1C and higher magnification in Figure S1C). These data clearly demonstrated constitutive differentiation-associated expression of CCL20 in normal human skin. In contrast, in HPV8-positive lesional areas of EV epidermis, CCL20 protein expression was only weak or completely lost (Figure 1EF and higher magnification in Figure S1D).

### C/EBPβ binds to the enhancer region of the *CCL20* gene in vivo

**Figure 2:**
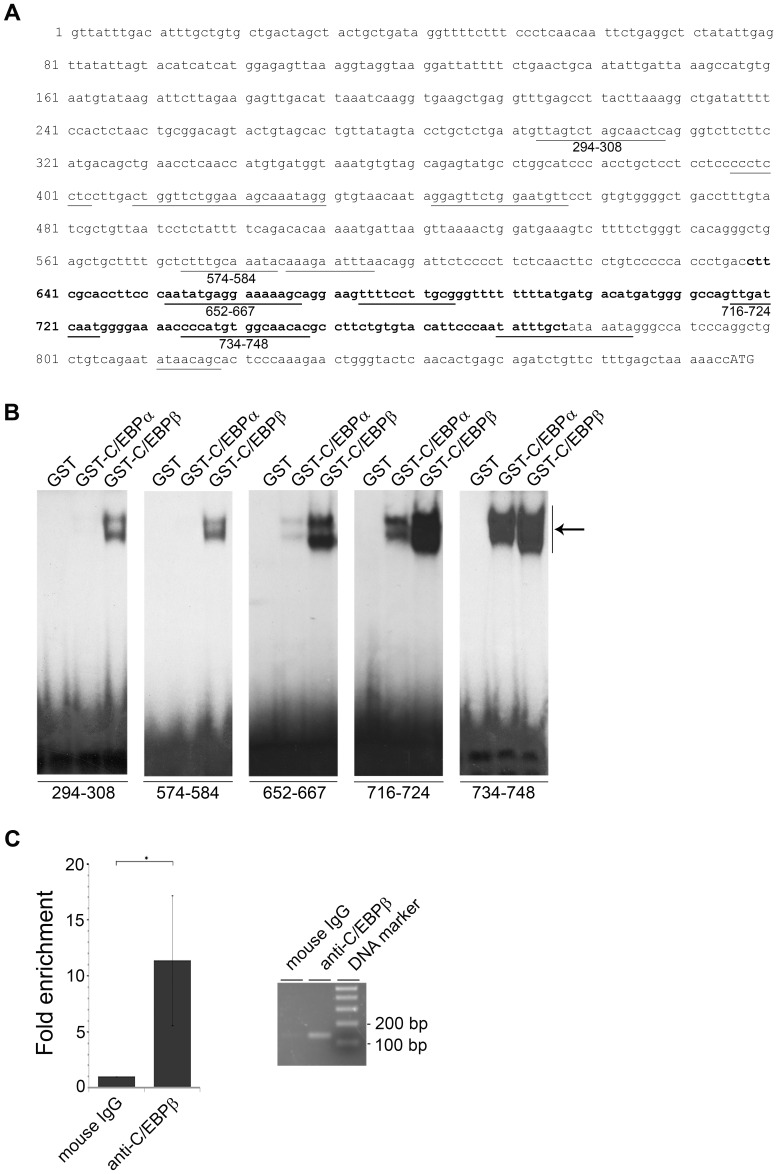
C/EBPβ binds to the enhancer region of CCL20 in vivo. (A) Nucleotide sequence of the human CCL20 promoter region with twelve putative C/EBP binding sites (underlined). Numbers below the underlined C/EBP binding sites mark the sequences, which display C/EBP DNA binding activity in EMSA. In bold is the DNA sequence tested for C/EBP binding in ChIP assay. (B) ^32^P-labeled oligonucleotides containing the respective C/EBP binding sites (nt 294–308, nt 574–584, nt 652–667, nt 716–724, nt 734–748) of the CCL20 promoter were incubated with 5 µg GST, GST-C/EBPα or GST-C/EBPβ fusion proteins and analyzed by EMSA. The arrow indicates complexes corresponding to C/EBP DNA binding activity. (C) Chromatin immunoprecipitation assay was performed using RTS3b cells transfected with the C/EBPβ expression vector. For precipitation anti-C/EBPβ (H-7) antibody was used. Genomic DNA was isolated, amplified by real-time PCR with primers specific for the nt 638–677 region of the CCL20 promoter (in bold). The amplicon was quantified (left panel) and visualized on an agarose gel (right panel). The amount of target DNA precipitated with the control antibody was set at 1. Shown are mean values ± SD from four experiments. The asterisk represents statistical significance, p = 0.02.

To gain more insight into *CCL20* gene regulation we performed in silico analysis of the *CCL20* 5′ regulatory region using the MatInspector software [35]. Twelve putative recognition sites for C/EBP factors were predicted (Figure 2A). This was of most interest, since these transcription factors are well known to regulate differentiation-specific gene expression in keratinocytes [36]–[39]. All putative sites were tested in EMSA. Five out of the twelve tested oligonucleotides were bound by bacterially expressed GST-tagged C/EBPα and C/EBPβ (Figure 2B). The two most promoter-proximal sites (nt 716–724 and nt 734–748) showed the highest binding affinities toward the C/EBP factors (Figure 2B). In all cases C/EBPβ displayed a higher DNA binding activity than C/EBPα.

Chromatin immunoprecipitation analysis confirmed binding of the C/EBPβ protein to the CCL20 promoter (nt 638–777) in vivo. Anti-C/EBPβ antibody-mediated precipitation led to a more than 11-fold enrichment of the DNA region comprising the promoter-proximal C/EBP binding sites as quantified by real-time PCR (p = 0.02) (Figure 2C). From these data the transcription factor C/EBPβ was regarded as a strong candidate regulating CCL20.

### C/EBPβ is expressed in differentiated layers of human epidermis and induces CCL20 in cultured human keratinocytes

**Figure 3:**
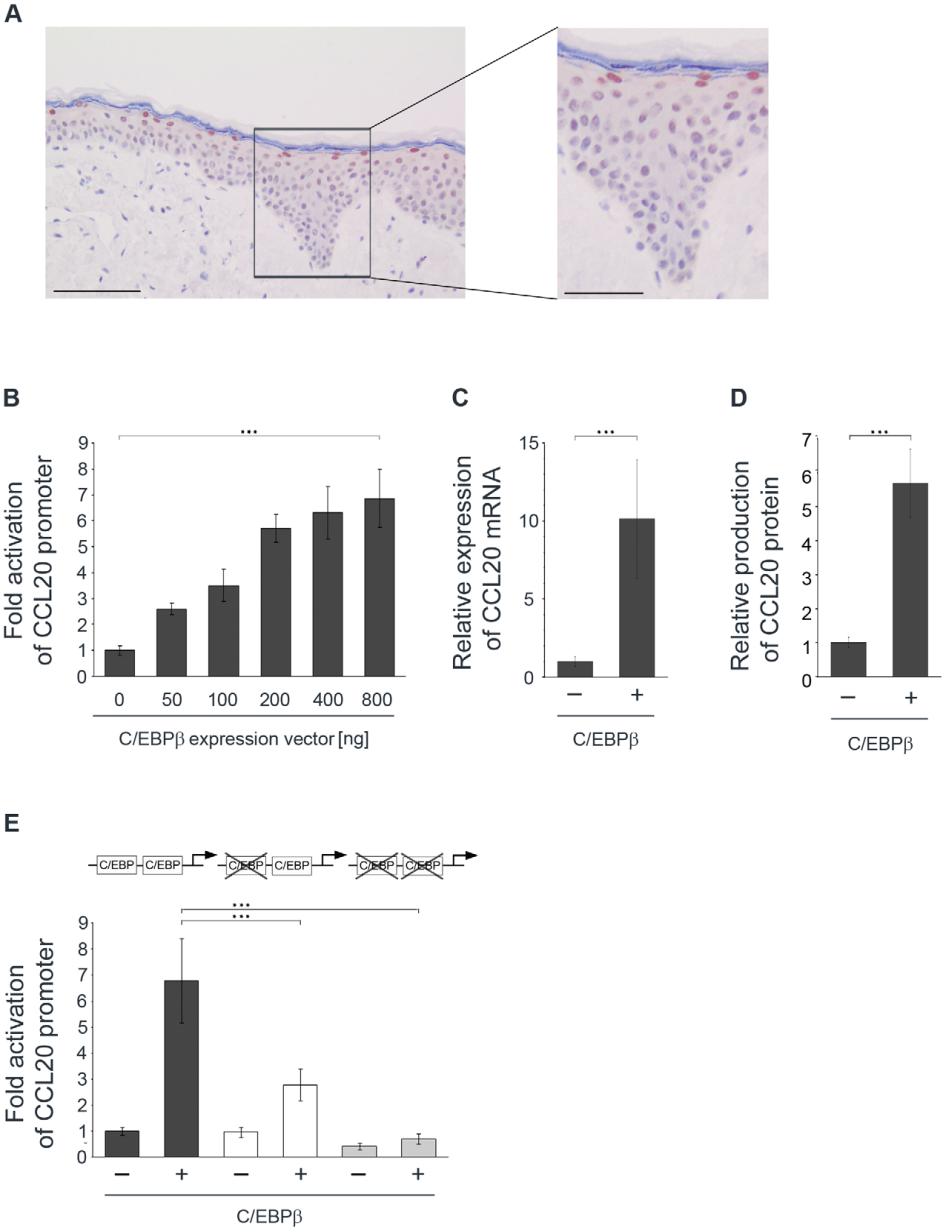
Differentiation-specific nuclear C/EBPβ expression in human epidermis and C/EBPβ-mediated induction of CCL20 expression in NHK. (A) Sections of normal human skin were stained using a polyclonal antibody against C/EBPβ (brown) and hematoxylin. A higher magnification of the complete epidermis is presented. Bars correspond to 100 µm and to 50 µm in the higher magnification. (B) NHK were co-transfected with the CCL20 promoter reporter construct and increasing amounts of C/EBPβ expression vector as indicated. Total amount of DNA was adjusted with the pcDNA3.1+ control vector. After 24 h the luciferase activity was measured and normalized to protein concentrations of the respective luciferase extracts. The normalized luciferase activity of the control transfection was set at 1. Shown are the values averaged from two transfections performed in triplicates ± SD. (C, D) RTS3b cells were transfected with C/EBPβ expression vector. After 24 h mRNA was isolated from the cells and quantified by real-time PCR for CCL20 in relation to GAPDH (C). Supernatants collected from these cell cultures were assessed for CCL20 protein expression by ELISA (D). Shown are the mean values ± SD from at least three independent experiments. Asterisks represent statistical significances, p<0.001. (E) Two promoter-proximal C/EBP binding sites are crucial for CCL20 activation. NHK were transfected with luciferase reporter constructs either under the control of the wild-type CCL20 promoter (black bars) or the CCL20 promoter mutated in one (white bars) or two (grey bars) proximal C/EBP binding sites and co-transfected with C/EBPβ (400 ng) expression vector. Total amount of DNA was adjusted with the pcDNA3.1+ control vector. After 24 h the luciferase activity was measured and normalized to protein concentration of the respective luciferase extracts. The normalized luciferase activity of the control transfection was set at 1. Shown are the values averaged from three transfections performed in triplicates ± SD. Asterisks represent statistical significances, p<0.0001.

To further evaluate the role of C/EBPβ as a putative inducer of CCL20 expression, we stained sections of normal human skin. As demonstrated in Figure 3A, C/EBPβ was strongly expressed in the nuclei of the uppermost differentiated keratinocytes. This pattern strikingly matched the pattern of CCL20 protein expression (Figure 1B, Figure 3A, and Figure S2) suggesting that C/EBPβ might be involved in *CCL20* gene activation in human epidermis.

To investigate whether the *CCL20* gene is specifically regulated through the C/EBPβ transcription factor, a luciferase reporter construct under the control of the 5′ regulatory region of CCL20 was used. Increasing amounts of C/EBPβ in primary normal human keratinocytes (NHK) activated the CCL20 luciferase reporter construct in a dose-dependent manner up to 7-fold (p<0.001) (Figure 3B).

In further experiments we investigated the regulation of the endogenous *CCL20* gene by C/EBPβ. To achieve higher transient transfection efficiencies, the keratinocyte RTS3b cell line was used. 24 h after transfection of the C/EBPβ expression vector, RNA and supernatants were analyzed by quantitative real-time PCR and ELISA, respectively. C/EBPβ expression caused a 10-fold up-regulation of endogenous CCL20 mRNA levels (p<0.001) (Figure 3C) and a 6-fold CCL20 protein induction (p<0.001) (Figure 3D) compared with the empty vector control.

### C/EBPβ activates the CCL20 promoter via two promoter-proximal binding sites

Next, the impact of the two promoter-proximal binding sites (nt 716–724 and 734–748) on activation of *CCL20* gene transcription was analyzed. Distinct mutations were introduced into these binding sites and DNA binding activity was tested in EMSA. Mutations in both binding sites led to an almost complete loss of C/EBP binding activity compared with the wild-type oligonucleotides (Figure S3). The same mutations were introduced into these binding sites in the CCL20 luciferase reporter construct by site-directed mutagenesis.

Transient transfections with CCL20 reporter constructs comprising the respective mutations were performed with NHK. Mutation of one C/EBP binding site (nt 716–724) reduced the C/EBPβ-induced promoter activity by 59% (p<0.0001). Additional mutation of the other C/EBP binding site (nt 734–748) completely abolished the C/EBPβ effect (p<0.0001) (Figure 3E) demonstrating the importance of the two promoter-proximal binding sites for C/EBPβ-mediated CCL20 induction.

### Endogenous C/EBPβ is a regulator of CCL20 in primary human keratinocytes

**Figure 4:**
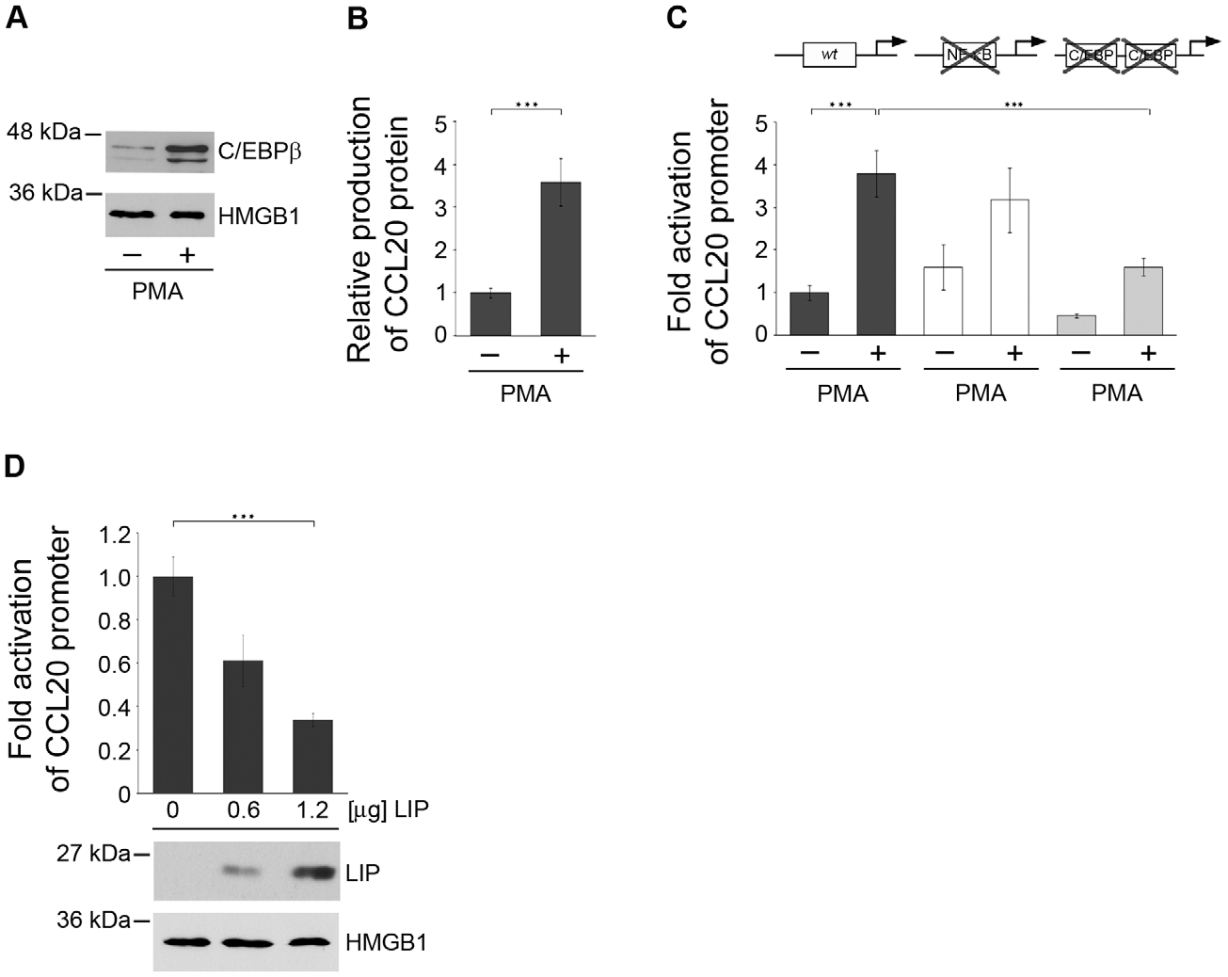
Endogenous C/EBPβ contributes to CCL20 expression in NHK. (A) Nuclear extracts isolated from NHK stimulated with 50 ng/ml PMA for 6 h were analyzed for C/EBPβ expression by Western blot. Anti-HMGB1 antibodies were used as a control. Shown is one representative experiment out of three. (B, C) NHK were transfected with luciferase reporter constructs either under the control of the wild-type CCL20 promoter (black bars), the CCL20 promoter containing mutations of the proximal NF-κB binding site (white bars) or mutations in the promoter-proximal C/EBP binding sites (grey bars). 24 h post-transfection the cells were stimulated with 50 ng/ml PMA or medium as a control. 24 h later expression of the CCL20 protein was measured by ELISA in supernatants of NHK transfected with the wild-type CCL20 promoter construct (B), while in all transfected cells luciferase activity was determined (C). (D) NHK were co-transfected with the wild-type CCL20 promoter and increasing amounts of the LIP expression vector (0.6, 1.2 µg). Total amount of DNA was adjusted with pcDNA3.1+ vector. LIP expression was investigated in Western blot analysis, HMGB1 was used as loading control. In all reporter gene assays luciferase activity was measured 24 h after transfection and normalized to protein concentrations of the respective luciferase extracts. The normalized luciferase activity of the control transfection was set at 1. Shown are the values averaged from two transfections performed in triplicates ± SD. Asterisks represent statistical significances, p<0.0001.

To evaluate the impact of endogenous C/EBPβ on CCL20 expression, we investigated basal levels of this transcription factor in NHK and after stimulation with PMA. PMA is a known activator of the NF-κB pathway as well as an inducer of C/EBPβ [40]–[44]. Nuclear extracts from NHK treated with 50 ng/ml PMA were prepared and subjected to Western blot analysis. Constitutive expression of C/EBPβ was observed in cultured NHK at low levels. Six hours after PMA stimulation nuclear C/EBPβ levels were strongly induced as compared with the constitutively expressed nuclear factor high mobility group protein B1 (HMGB1, [45]) (Figure 4A).

Supernatants from PMA-stimulated NHK were investigated for CCL20 protein levels by ELISA. CCL20 protein was induced about 3.5-fold as measured in the supernatants of the stimulated keratinocytes (p<0.0001) (Figure 4B). CCL20 protein levels ranged between 460–630 pg/ml (data not shown). Using the CCL20 reporter construct we analyzed the influence of PMA stimulation on transcriptional CCL20 regulation in NHK. CCL20 promoter activity was enhanced 3.5- to 4-fold by stimulation with PMA in NHK (p<0.0001) (Figure 4C). Results obtained with the CCL20 reporter assay corresponded well to the protein inducibility by PMA. Mutation of the promoter proximal NF-κB binding site within the CCL20 regulatory region [30], [31] reduced PMA-inducible promoter activity only by 16%, whereas mutations in both C/EBP binding sites reduced the stimulatory effect of PMA on CCL20 promoter activity by 58% (p<0.0001) (Figure 4C). This was in strong contrast to the results obtained with TNF-α and poly(I∶C) in NHK mediating activation of the CCL20 promoter exclusively through the previously described NF-κB site [31] (Figure S4).

To determine the impact of endogenous C/EBPβ expression on CCL20 regulation in NHK, a dominant-negative version of C/EBPβ (LIP) [46] was introduced by transient transfection. LIP expression was verified by Western blot analysis, HMGB1 was again used as a loading control. As a consequence, the basal activity of the co-transfected CCL20 reporter gene was suppressed by LIP in a dose-dependent manner (p<0.0001) (Figure 4D). Taken together these data demonstrated for the first time that the transcription factor C/EBPβ is an important regulator of CCL20 expression in human keratinocytes.

### HPV8 E7 protein binds to C/EBPβ and suppresses C/EBPβ-induced expression of CCL20

**Figure 5:**
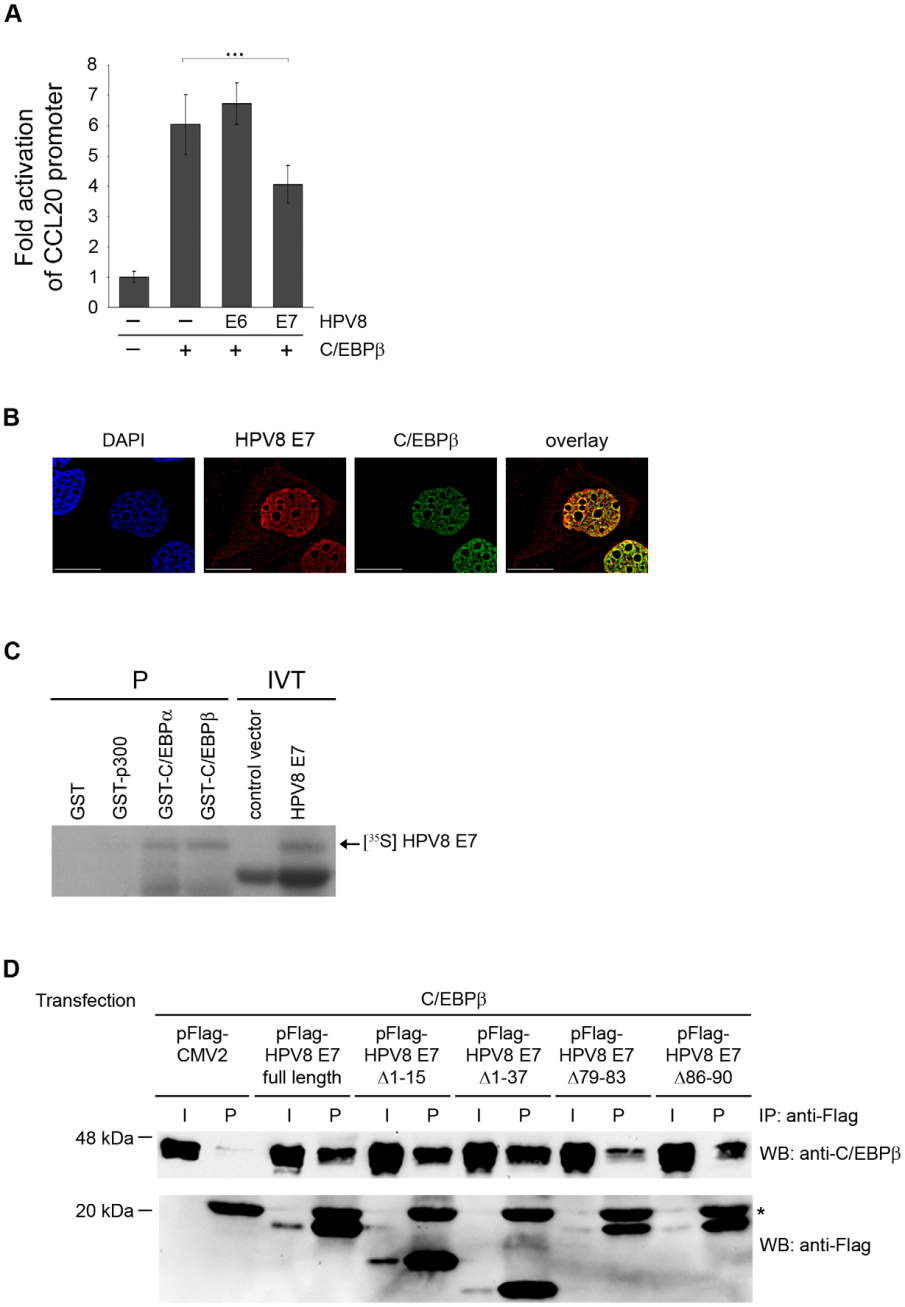
HPV8 E7 directly interacts with C/EBPβ and suppresses C/EBPβ-induced activation of the CCL20 promoter. (A) NHK were transfected with CCL20 promoter luciferase construct (0.5 µg) and C/EBPβ (0.4 µg) in the presence or absence of HPV8 E6 (0.8 µg) or HPV8 E7 (0.8 µg) pcDNA3.1+ expression vectors. Total amount of DNA was adjusted with empty vector. After 24 h the luciferase activity was measured and normalized to protein concentration of the respective luciferase extract. The normalized luciferase activity of the control transfection was set at 1. Transfections were conducted in triplicates. Shown are mean values from three independent experiments ± SD. Asterisks represent statistical significance, p<0.001. (B) HPV8 E7 co-localizes with C/EBPβ in the keratinocyte nucleus. RTS3b cells seeded on glass coverslips were co-transfected with Flag-HPV8 E7 and ECFP-C/EBPβ expression vectors and stained with DAPI (blue, first panel) as well as anti-Flag antibody (red, second panel). ECFP-C/EBPβ is shown in third panel (green). Cells were analyzed by deconvolution fluorescence microscopy. The overlays and co-localization (yellow) are displayed in the fourth panel. Bars correspond to 20 µm. (C) In pull-down assays GST, GST-p300, GST-C/EBPα or GST-C/EBPβ were incubated with in vitro-translated HPV8 E7 protein (IVT, input) and precipitated (P) by glutathione Sepharose beads. pcDNA3.1+ vector (control vector) was used as a control for HPV8 E7 in vitro translation. Proteins were visualized by SDS-PAGE and autoradiography. (D) C33A cells were transfected with pFlag-CMV2, the Flag-tagged HPV8 E7 construct or deletion mutants thereof and co-transfected with the C/EBPβ expression vector. After 24 h cell lysates were prepared and precipitated with anti-Flag agarose beads (IP). The precipitates (P) and input (I, 10 µl of the lysates) were analyzed with anti-C/EBPβ or anti-Flag antibodies by Western blot (WB). The anti-Flag antibody light chain present in the precipitates is marked with a star (*).

Next, we studied the impact of HPV8 oncoproteins on C/EBPβ-mediated CCL20 induction. Transient co-transfection experiments were performed in NHK. HPV8 E7 expression significantly reduced the C/EBPβ-induced CCL20 promoter activation (p<0.001) (Figure 5A). The level of repression in these transient transfection experiments was limited by the fact that not all C/EBPβ-transfected cells were co-transfected with HPV8 E7 (co-transfection efficiency of both factors 50–60%). In contrast, expression of the HPV8 E6 protein did not interfere with the C/EBPβ-mediated activation of the CCL20 promoter (Figure 5A). This demonstrated that C/EBPβ-mediated CCL20 induction is specifically suppressed by the HPV8 E7 but not the E6 protein.

To gain more insight into the mechanism of suppression co-localization studies were performed. Flag-tagged HPV8 E7 was detected at lower levels in the cytoplasm of transfected keratinocytes and stronger expression was observed in the nucleus, where it co-localized with the nuclear C/EBPβ transcription factor (Figure 5B, fourth panel).

A potential physical interaction between HPV8 E7 and C/EBPβ was assessed by in vitro and in vivo binding assays. In vitro translated and radioactively labeled HPV8 E7 was precipitated by GST-C/EBPβ and GST-C/EBPα demonstrating that the E7 protein interacts with both C/EBP factors in vitro. Neither GST protein alone, nor GST-p300, which was used as a control, precipitated HPV8 E7 (Figure 5C). Vice versa, bacterially expressed GST-HPV8 E7 but not GST precipitated C/EBPβ from cellular lysates of transfected C33A cells (data not shown). The in vivo interaction between HPV8 E7 and C/EBPβ was investigated by co-immunoprecipitation assays in C33A cells co-transfected with C/EBPβ and Flag-tagged HPV8 E7 or empty pFlag-CMV2 control vector. As shown in Figure 5D, C/EBPβ protein was present in all input cellular extracts and was strongly co-precipitated by the Flag-HPV8 E7 protein as compared with Flag alone. The co-immunoprecipitation assays confirmed an interaction between HPV8 E7 and C/EBPβ in vivo. HPV8 E7 mutants deleted from the N-terminus (pFlag-HPV8 E7Δ1-15, E7Δ1-37) still displayed strong binding to C/EBPβ (Figure 5D) and significantly suppressed the C/EBPβ-inducible transactivation of the CCL20 promoter (Figure S5A). HPV8 E7 mutants, in which the putative C-terminal zinc finger domain was deleted, were not detectable (data not shown), presumably due to instability as previously described for the HPV16 E7 protein [47]. Deletion mutants within the loop region of the putative zinc finger (pFlag-HPV8 E7Δ79-83 and E7Δ86-90) were stably expressed (Figure S5B). Only the HPV8 E7 deletion mutant lacking amino acids 79–83 (FQELL) lost the ability to significantly suppress C/EBPβ-inducible CCL20 transactivation (Figure S5A). Correspondingly, the same mutant displayed the weakest binding to C/EBPβ (Figure 5D). Thus, the FQELL sequence within the loop region of the C-terminal zinc finger of HPV8 E7 plays an important role for binding to C/EBPβ as well as suppression of C/EBPβ-mediated CCL20 induction.

### HPV8 E7 protein interferes with C/EBPβ binding to the CCL20 promoter in vivo

**Figure 6:**
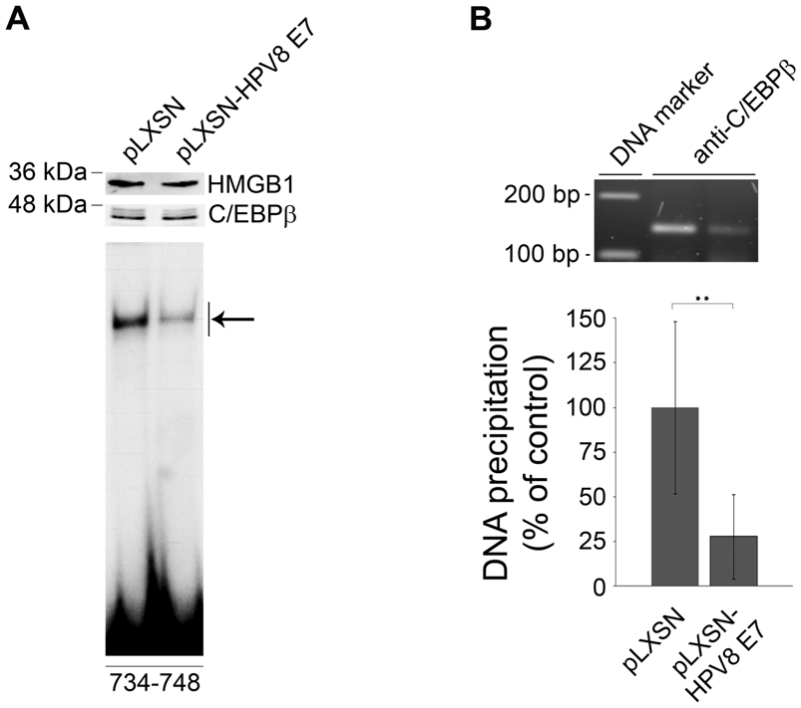
HPV8 E7 interferes with binding of C/EBPβ to the CCL20 promoter. (A) Nuclear extracts from HaCaT cells stably expressing HPV8 E7 (pLXSN-HPV8 E7) and corresponding control cells (pLXSN) were analyzed by Western blot for C/EBPβ protein and HMGB1 expression (upper panels). Identical amounts of the respective nuclear extracts were used for EMSA using the ^32^P-labeled oligonucleotides (nt 734–748) containing the C/EBP binding site in the CCL20 promoter (lower panel). The complex corresponding to endogenous C/EBP binding activity within the CCL20 promoter is indicated by an arrow. (B) The same cells were used for chromatin immunoprecipitation. Protein-genomic DNA complexes were precipitated with anti-C/EBPβ antibody. DNA was isolated, amplified by real-time PCR with primers specific for the nt 638–677 region of the CCL20 promoter. The amplicon was quantified (lower panel) and visualized on an agarose gel (upper panel). The amount of target DNA precipitated from the pLXSN control cells was set at 100%. The mean values ± SD from three independent experiments are presented. Asterisks represent statistical significance, p = 0.008.

To shed more light on the mechanism of how HPV8 E7 interfered with C/EBPβ function, HPV8 E7 was stably expressed in HaCaT cells by retroviral gene transfer using the pLXSN vector system. Viral gene expression was confirmed by RT-PCR (Figure S6A). In nuclear extracts of HPV8 E7 expressing HaCaT and control pLXSN cells C/EBPβ was detected at similar levels by Western blot analysis (Figure 6A, upper panel). Normalized to the HMGB1 loading control, C/EBPβ protein levels were 1.15-fold higher in HPV8 E7 expressing cells. Notably, when the same extracts were analyzed in EMSA using the C/EBPβ binding site (nt 734–748) of the CCL20 promoter, binding activity was strongly diminished (Figure 6A, lower panel). Quantification of shifted bands in EMSA revealed a more than 50% reduction of DNA binding activity as compared with C/EBPβ protein expression. Also in vivo, C/EBPβ binding to the CCL20 promoter (nt 638–777) was strongly reduced (by 72%) in HPV8 E7 expressing HaCaT cells as demonstrated by chromatin immunoprecipitation analysis (Figure 6B). These data clearly demonstrated that the HPV8 E7 protein does not lead to degradation of the cellular transcription factor C/EBPβ but interferes with its binding to the CCL20 promoter.

### The HPV8 E7 but not E6 protein suppresses CCL20 expression in normal human keratinocytes as well as Langerhans cell migration

**Figure 7:**
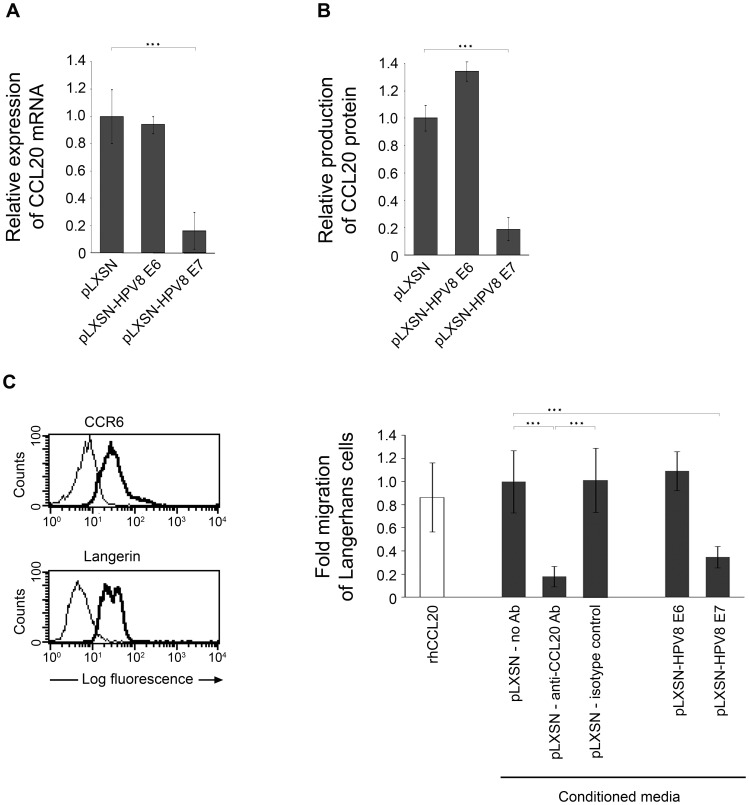
HPV8 E7 suppresses CCL20 expression and Langerhans cell migration. CCL20 mRNA (A) and protein (B) levels were quantified in NHK stably expressing HPV8 E6 (pLXSN-HPV8 E6) or E7 (pLXSN-HPV8 E7) oncogenes. The amount of CCL20 mRNA (in relation to β-actin as measured by quantitative real-time PCR) or protein in control cells was set at 1. CCL20 protein levels in supernatants collected from E6 or E7 expressing keratinocytes and corresponding control cells (pLXSN) were determined by ELISA. Measurements represent the mean values from three independent retroviral infections ± SD. Asterisks represent statistical significance, p<0.001. (C) Migration of langerin- and CCR6-positive (left panel) Langerhans cells was assessed in response to conditioned media collected from pLXSN control NHK in the presence or absence of anti-CCL20 antibody (Ab) or respective isotype control Ab and from NHK stably expressing HPV8 E6 (pLXSN-HPV8 E6) or E7 (pLXSN-HPV8 E7) oncogenes (right panel). Recombinant human CCL20 (rhCCL20) was used as a positive control. After 24 h transmigrated cells were counted. Data were collected in triplicates from at least two independent experiments using conditioned media from two independent retroviral infections. Shown are the mean values ± SD. Asterisks represent statistical significances, p<0.0001.

NHK stably expressing HPV8 E6 or HPV8 E7 (Figure S6B) were generated by retroviral gene transfer similar to HaCaT cells. CCL20 mRNA levels were quantified by real-time PCR, protein levels were determined in cell supernatants by ELISA. In HPV8 E7 expressing cells from three independent retroviral infections CCL20 was reduced by 80%, at both the mRNA and protein levels as compared with the corresponding vector control cells (Figure 7AB). Similar results were obtained for HPV8 E7 expressing HaCaT cells (Figure S6CD). In contrast, expression of CCL20 was not reduced in HPV8 E6 expressing NHK (Figure 7AB).

CCL20 potently stimulates Langerhans cell migration, which was also observed with conditioned media from pLXSN control NHK. Migration experiments performed with these conditioned media in the presence of neutralizing CCL20 antibodies reduced Langerhans cell migration by 82% as compared with the isotype control antibody (Figure 7C, right panel). This indicated that CCL20 is the major factor present in the conditioned media of our keratinocytes and that other factors might play only a minor role for this activity. To investigate the impact of HPV8 oncoproteins on Langerhans cell migration, supernatants of NHK stably expressing HPV8 E6 or HPV8 E7 were also used in transwell migration assays. Only conditioned media from HPV8 E7, but not from HPV8 E6 expressing NHK, reduced migration of Langerhans cells by more than 65% as compared with conditioned media from pLXSN control NHK (Figure 7C, right panel). These data demonstrated that the HPV8 E7 but not the E6 protein strongly suppresses expression of CCL20 in human keratinocytes as well as Langerhans cell migration.

## Discussion

**Figure 8:**
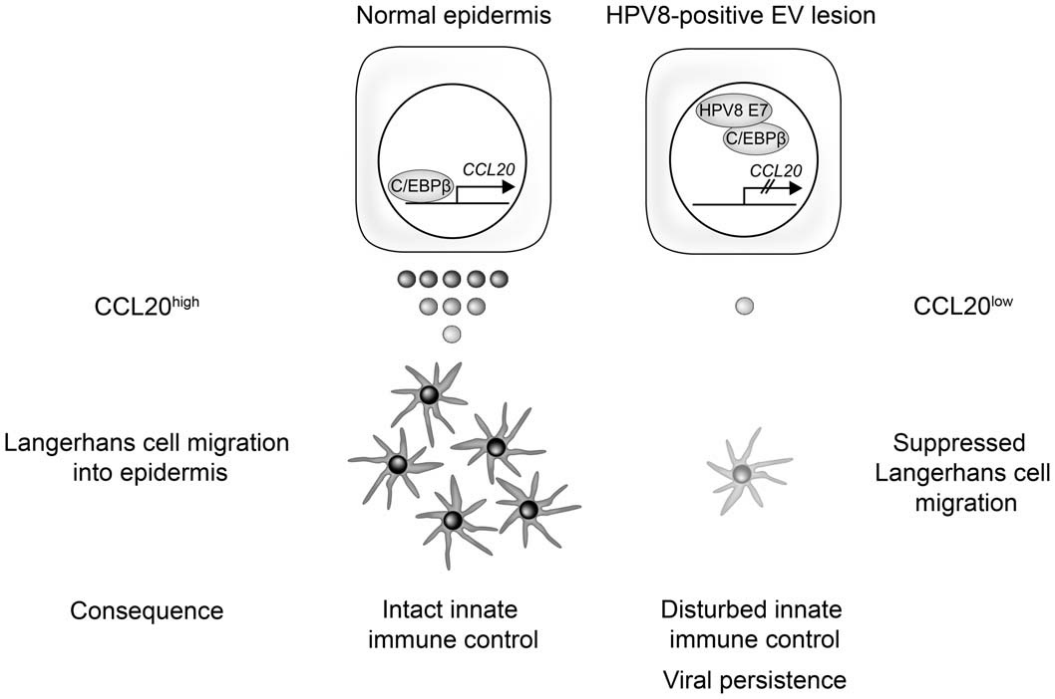
Schematic presentation of the molecular mechanism how human papillomavirus type 8 interferes with a novel C/EBPβ-mediated mechanism of keratinocyte CCL20 chemokine expression and Langerhans cell migration.

Our study shows for the first time that the Langerhans cell chemoattractant protein CCL20 is virtually absent in skin lesions of EV patients and we confirm a strong reduction of Langerhans cell numbers in these lesions. We have identified C/EBPβ as a novel key regulator of CCL20 expression in human keratinocytes. This mechanism is specifically targeted by the HPV8 E7 protein. As a consequence, HPV8 E7 expressing keratinocytes produce only low amounts of CCL20 and their capacity to attract Langerhans cells is strongly impaired. Our data provide evidence for a novel HPV-driven molecular mechanism, which may enable the virus to evade detection by the local immune system and allow its persistence in the epithelium (Figure 8).

It is well established that epidermal Langerhans cells play a key role in initiating and regulating adaptive immune responses in skin [20]–[22]. Their precursor cells express CCR6, a chemokine receptor for CCL20 directing them into the epidermis along a chemotactic gradient [20]–[22]. Applying sensitive immunohistochemical analyses, this study shows that CCL20 is specifically expressed in the uppermost differentiated layers of normal human epidermis. So far differentiation-associated mechanisms of CCL20 regulation were unknown. Our data demonstrate that CCL20 expression in human epidermis spatially converges with nuclear expression of the transcription factor C/EBPβ. Detailed experiments unraveled C/EBPβ as a potent functional regulator of CCL20 mRNA and protein expression in human keratinocytes. Functional inhibition of endogenous C/EBPβ activity in NHK with a dominant-negative C/EBPβ mutant reduced CCL20 promoter activity. Conversely, overexpression of C/EBPβ strongly increased its activity and led to endogenous CCL20 mRNA and protein induction.

We found that C/EBPβ strongly binds to two promoter-proximal C/EBP binding sites by EMSA and chromatin immunoprecipitation assays. One of these sites had previously been studied but turned out not to mediate IL-1β-inducible CCL20 reporter gene activation as investigated in the intestinal cell line Caco-2 [31]. Mutational analyses in our study clearly demonstrated that both binding sites are required to mediate full C/EBPβ-induced transcriptional activation of the *CCL20* promoter in human keratinocytes.

So far NF-κB had been described as a transcriptional regulator of CCL20. An NF-κB site located between the two C/EBP binding sites found in this study is crucial for TNF-α-, poly(I∶C)- (shown in this and in other studies), IL-1α-, or IL-1β-inducible *CCL20* gene expression [29]–[31], [48]–[50]. PMA, a strong activator of PKC leads to NF-κB signaling [40], [41] as well as C/EBP up-regulation [42]–[44] (as also shown here) and can induce differentiation-associated genes in keratinocytes [36]. Of note, our data show that the C/EBP binding sites strongly contributed to PMA-mediated CCL20 promoter activation aside from the well-characterized NF-κB binding site.

From these and our novel data it can be concluded that there are two major signaling pathways, which drive inducible CCL20 expression. While the NF-κB pathway governs inflammatory CCL20 induction, our newly identified C/EBPβ-dependent regulatory mechanism is proposed to be most relevant for differentiation-associated CCL20 induction in human keratinocytes. This hypothesis is strongly supported by the fact that C/EBPβ shows highest nuclear expression in the differentiated granular layers of normal human epidermis.

As demonstrated in this study, EV skin lesions harboring only HPV8 or co-infected with HPV8 and 5, showed strongly reduced numbers of Langerhans cells and displayed only low CCL20 protein levels or the chemokine was virtually absent. In our in vitro experiments, HPV8 E7 interfered with CCL20 promoter activation induced by ectopic C/EBPβ overexpression. Moreover, HPV8 E7 selectively suppressed constitutive CCL20 protein and mRNA expression in cultured NHK displaying low endogenous C/EBPβ expression levels. As a consequence, the capacity of keratinocytes to attract Langerhans cells was potently suppressed when HPV8 E7 was expressed. This corresponded well to our in vivo observation that EV lesions with productive HPV8 infection are poorly (re-)populated with Langerhans cells. It can be assumed that decreased immunosurveillance in these lesional areas may allow the virus to persist and promote oncogenic disease progression.

Interestingly, inhibition of CCL20 promoter activation, mRNA and protein expression as well as reduced Langerhans cell attraction was only seen in the context of the HPV8 E7 but not the E6 oncoprotein. While it is well established that the genus beta HPV E6 oncoprotein protects infected keratinocytes from apoptosis [51], our data provide evidence that the HPV8 E7 protein interferes with innate immune control. Thus both, the E6 and the E7 oncoproteins, are proposed to have important roles during the process of HPV-associated skin carcinogenesis.

While an indirect effect of the mucosal high-risk HPV16 E7 protein on C/EBPα-mediated differentiation and proliferation arrest has previously been reported [52], HPV-mediated interference with CCL20 expression has as yet only been investigated in the context of the inflammatory poly(I∶C)-induced NF-κB pathway. E6 and E7 proteins from the mucosal high-risk type HPV16 as well as the low-risk types HPV6 and 11 were shown to suppress NF-κB-dependent CCL20 induction [53]. In line with this, E6 and E7 silencing in the HPV16-positive cervical cancer cell line SiHa restored nuclear expression of NF-κB subunits and increased CCL20 expression [54]. Thus, NF-κB has clearly been defined as a target of the mucosal HPV oncoproteins. Our study adds a novel pathway to the list of HPV oncoprotein targets, in this case the HPV8 E7 oncoprotein.

Similar to the genital high-risk HPV16 E7 protein, HPV8 E7 was expressed at lower levels in the cytoplasm of keratinocytes and predominantly found in their nuclei [55]. Our data demonstrate that in the nucleus HPV8 E7 co-localizes with the cellular transcription factor C/EBPβ. Further analyses clarified the molecular basis of the HPV8 E7-mediated suppression of C/EBPβ-dependent CCL20 induction. Co-immunoprecipitation assays documented a specific in vivo interaction between HPV8 E7 and C/EBPβ. Deletion analysis of the HPV8 E7 protein revealed that the C-terminal FQELL sequence at positions 79–83 within the loop of the putative zinc finger plays an important role for C/EBPβ binding and inhibition of its ability to transactivate the CCL20 promoter. Of note, also HPV16 E7 has been described to bind several cellular proteins within the corresponding C-terminal loop region [56], [57]. However, since the amino acid sequences at these positions differ between HPV8 E7 (FQELL) and HPV16 E7 (LEDLL), it cannot be deduced whether or not the genus alpha HPV16 E7 protein also binds C/EBPβ within this region.

Assays with in vitro translated and bacterially expressed proteins argue for a direct interaction between HPV8 E7 and the cellular factor C/EBPβ. The interaction between both factors was highly specific since E7 neither bound to the nuclear protein HMGB1 (data not shown) nor to p300, a transcriptional co-activator recently found to be a target of the HPV8 E6 protein [58], [59]. This supports the notion that HPV8 E7 actively manipulates host cell functions via direct interactions with cellular factors, such as retinoblastoma protein [60], TATA-binding protein or TBP-associated factors [61] and Smad proteins [62]. Results of the present study have unequivocally identified C/EBPβ as a novel cellular interaction partner for HPV8 E7. Our analyses further showed that the HPV8 E7 protein did not lead to C/EBPβ degradation but interfered with its binding to the C/EBPβ target sequence within the CCL20 promoter in chromatin immunoprecipitation assays and EMSA.

This is the first study providing evidence for a key role of the transcription factor C/EBPβ in regulation of the Langerhans cell chemoattracting protein CCL20. We show that this molecular mechanism is specifically targeted by the HPV8 E7 oncoprotein. As a consequence HPV8 E7 expressing keratinocytes display strongly reduced activity to attract Langerhans cells. In line with these data, HPV8-positive skin lesions from EV patients showed strongly decreased levels of CCL20 expression and were associated with lower numbers of Langerhans cells. This may prevent efficient viral antigen presentation and allow HPV8 to escape from local immunosurveillance.

In summary, our study not only identified a novel cellular mechanism important for skin immunity but also a pathophysiological mechanism that can explain viral persistence in the epidermis, which is a major prerequisite for virus-induced tumor progression.

## Materials and Methods

### Ethics statement

The study was conducted according to the principles expressed in the Declaration of Helsinki. The study was approved by the Bioethics Committee at the Medical University of Warsaw, Poland, the Ethics Committees of the Medical Faculty of the University of Cologne and the Saarland University at the Saarland Ärztekammer. Written informed consent was provided by all study participants.

### Immunohistochemical staining and HPV genotyping

Anonymized skin specimens from EV lesions fixed in 4% neutral buffered formalin, processed routinely and embedded in paraffin were obtained from the Department of Dermatology, Medical University of Warsaw, Poland. The retrospective study was conducted according to the Declaration of Helsinki Principles. These lesions were screened for the presence of genus beta HPV by PCR and reverse hybridization according to the manufacturer's protocol (DDL, Voorburg, the Netherlands) [63] and two HPV8-containing lesions were selected for immunohistochemistry. As controls, anonymized paraffin-embedded skin specimens from ten patients were taken from the pathology archive, University of Cologne, Germany. Written informed consent was given and the protocol was approved by the local Ethics Committee. Antigen retrieval was performed by heating the sections either in citrate buffer pH 6.0 (langerin) or EDTA buffer pH 9.0 (C/EBPβ, CCL20) at 95°C for 15 min. For immunohistochemistry rabbit anti-C/EBPβ polyclonal antibody C-19 (1∶200, Santa Cruz Biotechnology, Heidelberg, Germany), rat anti-CD207 (langerin) antibody clone 929F3.01 (1∶50, Dendritics, Lyon, France) and goat anti-CCL20 polyclonal antibody AF360 (1∶200, R&D Systems, South Beloit, IL) were used. Paraffin sections were incubated with the indicated antibodies overnight followed by biotin-conjugated secondary antibodies. Finally, avidin-biotin complex was added and amino-ethyl-carpool was used as chromomeric substrate (Dao, Hamburg, Germany). Slides were evaluated with an Aristophanes microscope (Leitz, Wetzlar, Germany). Blocking experiments were performed by preincubation of the primary CCL20 antibody with recombinant CCL20 protein (PeproTech, Rockyhill, NJ). E4-specfic antibodies were raised in rabbits using a purified Maltose Binding Protein-E4 fusion protein as antigen. Immunofluorescence staining and the detection of genome amplification by FISH were carried out according to standard protocols [64], [65] except that antigen exposure was carried out using ‘Antigen Unmasking Solution (Vector Laboratories H3300)’ by heating in a pressure cooker for 10 minutes at 121°C.

### Plasmids, mutagenesis and generation of fusion proteins

HPV8 E6, HPV8 E7, C/EBPβ, the C-terminal portion of C/EBPβ representing the dominant-negative mutant LIP in pcDNA3.1+, pECFP-C/EBPβ (starting from the second ATG) [66]–[68], pLXSN-HPV8 E6, pLXSN-HPV8 E7 [67] and CMV-EGFP [69] were reported previously. pCMV-Flag2-HPV8 E7 was kindly provided by Dr. M. May. The deletion mutants pFlag-HPV8 E7Δ1-15, E7Δ1-37 were amplified using the forward PCR primers 5′-AAGCTTAGTGAGATACAACCTGAAGTGTTACCAGTTGA-3′ and 5′-AAGCTTGAAACGGAGGAGGAGCTAGACATC-3′, respectively, and the reverse primer 5′-TCTAGATTATGATCCGCCATGTTTGCAGTTACC-3′ and cloned into the pCMV-Flag2 vector (Sigma-Aldrich, Taufkirchen, Germany) within HindIII and XbaI sites. E7Δ79-83 and E7Δ86-90 were generated from pCMV-Flag2-HPV8 E7 using mutagenic primers 5′-TTCGGGTATCAGGACCTTCAGAGACCTACAGC-3′ and 5′-GACCTTTCAAGAATTGCTGTTCAGATGTCCTGAGTGCC-3′, respectively, and the QuickChange site-directed mutagenesis kit (Agilent Technologies, Boeblingen, Germany). All constructs were verified by sequencing.

The luciferase reporter vector pGL3-CCL20 comprised the regulatory region of the *CCL20* gene (GenBank accession number AY150053) [31]. The pGL3-CCL20 NF-κBmut luciferase reporter construct contained mutations within the proximal NF-κB binding site at nucleotide positions 726–735 of the CCL20 promoter [31]. pGL3-CCL20 C/EBPmut contained four point mutations within the C/EBP binding site (TTGATCAAT changed to TTCAACGAG) within nucleotide positions 716–724 of the CCL20 promoter region [31]. The pGL3-CCL20 2×C/EBPmut plasmid with two mutated proximal C/EBP-binding sites was generated on the basis of pGL3-CCL20 C/EBPmut using QuickChange XL site-directed mutagenesis kit (Stratagene, La Jolla, CA) according to the manufacturer's instructions. Three point mutations (underlined) G>A, A>C, C>G within the C/EBP binding site at nucleotide positions 734–748 were introduced using the mutagenic primer 5′-GGGGAAAACCCCATGTGACCAGACGCCTTCTGTGTA-3′. Presence of the mutations was verified by DNA sequencing.

GST-C/EBPα, GST-C/EBPβ, GST-p300 histone acetyltransferase or GST-HPV8 E7 (in pGEX-2T) were expressed in *E. coli* and purified with glutathione-Sepharose 4B (GE Healthcare, Germany) [62], [66]. Protein concentrations were determined by Bradford assay.

### Cell culture, retroviral infection and Langerhans cell migration assay

NHK were cultured in supplemented KBM-2 medium (Lonza, Basel, Switzerland) [70]. The HPV-negative skin squamous cell carcinoma-derived RTS3b cell line [71] was grown as reported previously [66]. The HPV-negative cervical carcinoma cell line C33A (HTB-31: American Type Culture Collection, Manassas, VA) and HaCaT cells were grown in Dulbecco's modified Eagle's medium with Glutamax I supplemented with 10% fetal calf serum, 100 U/ml penicillin, 0.1 mg/ml streptomycin and 1 mM sodium pyruvate (all from Invitrogen, Karlsruhe, Germany). NHK or HaCaT stably expressing HPV8 early proteins were generated by retroviral gene transfer as previously described [67], [72] using pLXSN-HPV8 E6 or pLXSN-HPV8 E7 or control pLXSN vectors. Viral oncogene expression was confirmed by RT-PCR (Figure S5AB). Langerhans cells were obtained from purified monocytes isolated from whole blood of healthy subjects (written informed consent was given and the protocol was approved by the local Ethics Committee) as described previously [73] and cultured under endotoxin-free conditions in RPMI supplemented with 10% FCS, 20 ng/ml TGF-β, 5 ng/ml IL-4 (both from PeproTech), 100 ng/ml Leukine (Berlex, Montville, NJ). At day 8, cells were stained for langerin and CCR6 (clones 929F3.01 and 11A9, both from BD Biosciences, Heidelberg). In parallel, Langerhans cells were seeded onto transwell chambers placed in 24-well plates (8 µm pore size, Corning Costar Corp, NY), stimulated with conditioned media from retrovirally infected NHK and their migration capacity was assessed after 24 h. Background migration in culture medium only was subtracted. Recombinant CCL20 protein (600 pg/ml) was used as a positive control. CCL20 polyclonal antibody AF360 (1 µg/ml, R&D Systems) or the respective isotype control antibody (AB-108-C, 1 µg/ml, R&D Systems) were used in neutralization experiments.

### Transient transfections, cellular lysates and reporter gene analysis

NHK were seeded into 6-well plates at a density of 2.5×10^5^ cells per well and transfected after 24 h with 2 µg of DNA and 6 µl of TransFast transfection reagent (Promega, Mannheim, Germany) according to the manufacturer's protocol. In transient transfections 0.5 µg of the reporter construct together with 0.05–0.8 µg of C/EBPβ, or 0.6, 1.2 µg LIP, 0.8 µg HPV8 E6 or E7 in pcDNA3.1+ were used and adjusted to 2 µg with the respective empty vectors. 24 h post-transfection cellular lysates were prepared as described previously [70] and assayed for luciferase activity using a Wallac 1420 Victor2 Multilabel Counter (Perkin Elmer, Waltham, MA). The values were normalized to the protein contents of the lysates. In some experiments, cells were stimulated with 50 ng/ml of PMA (Sigma-Aldrich Chemicals) 24 h post-transfection and luciferase activity was measured 24 h later.

RTS3b cells were transfected with FuGene 6 (Roche, Mannheim, Germany) or Lipofectamine 2000 (Invitrogen) transfection reagents according to the manufacturers' guidelines. In RTS3b reporter assays 0.2 µg of C/EBPβ and 0.5 µg HPV8 E7 or its deletion mutants in pCMV-Flag2 were co-transfected in 6-well plates and the total amount of DNA was adjusted to 2 µg with respective control vectors. Transfection efficiencies in these cells reached up to 65%, co-transfection efficiency of HPV8 E7 in C/EBPβ expressing cells reached 50–60%.

C33A cells were seeded at a density of 3.16×10^6^ cells per 10-cm dish and transfected after 24 h with 15 µg of C/EBPβ or co-transfected with 7.5 µg of pCMV-Flag2, pFlag-HPV8 E7 or deletion mutants thereof together with 7.5 µg of C/EBPβ using the calcium phosphate method according to a standard procedure [74]. After 48 h the cells were harvested using trypsin, washed twice with PBS, lysed in a buffer containing PBS, 0.1% IGEPAL CA-630 (Sigma-Aldrich), 1 mM EDTA pH 8.0 (Sigma-Aldrich) and one protease inhibitor cocktail tablet (Complete Mini, Roche) per 10 ml and used for interaction assays.

### Co-localization, protein immunoprecipitation and Western blot analysis

RTS3b were grown on glass coverslips and co-transfected with 1 µg pFlag-HPV8 E7 and 1 µg pECFP-C/EBPβ using TransFast. After 24 h cells were washed, fixed and permeabilized as previously described [70]. HPV8 E7 was detected by mouse anti-Flag antibody (clone M5, Sigma-Aldrich Chemicals) diluted 1∶300 followed by Cy3-conjugated secondary antibody (Dianova, Hamburg, Germany). For visualization fluorescence deconvolution microscopy (Leica DMI 6000B microscope, MMAF 1.3 AutoQuant Deconvolution software, Leica, Wetzlar Germany) was used to study co-localization [75].

In vitro translated ^35^S-labeled HPV8 E7 (TNT T7 Coupled Reticulocyte Lysate System, Promega) or cellular extracts containing C/EBPβ (data not shown) were incubated with GST-fusion proteins and precipitated by glutathione Sepharose 4B. 800 µl C33A cellular extracts containing Flag-HPV8 E7 or deletion mutants thereof and C/EBPβ were precipitated using Flag Agarose (Flag M2 Affinity Gel, Sigma-Aldrich Chemicals), washed with PBS containing 0.5 M NaCl (Sigma-Aldrich), 0.1% IGEPAL CA-630, 1 mM EDTA pH 8.0. The precipitates as well as the nuclear extracts from NHK stimulated with 50 ng/ml of PMA for 6 h [76] or nuclear extracts from retrovirally infected HaCaT cells were subjected to SDS-PAGE and Western blotting as previously described [66]. Rabbit polyclonal anti-C/EBPβ antibody (C-19) recognizing also the 20 kDa dominant-negative version of C/EBPβ (LIP), mouse monoclonal anti-C/EBPβ antibody (400 ng/ml, H-7, sc-7962, Santa Cruz Biotechnology), mouse anti-Flag (clone M5), mouse anti-HMGB1 (clone 115603, R&D Systems), mouse anti-actin (500 ng/ml, clone C4, 1501R, Chemicon, Temecula, CA) as well as horseradish peroxidase-conjugated goat anti-rabbit (400 ng/ml, 111-035-047, Dianova) or goat anti-mouse (400 ng/ml, 115-035-072, Dianova) antibodies were used in Western blot analysis followed by chemiluminescence detection (Roche) according to the manufacturer's instructions. ChemiDoc XRS+ Molecular Imager and the Quantity One analysis software (both BioRad, Philadelphia, PA) were used for quantification. ^35^S-methionine-labeled proteins were visualized by autoradiography. According to the manufacturer's description, reticulocyte lysates contain globin presenting as bands at 10–15 kDa.

### CCL20 ELISA

RTS3b cells were seeded at a density of 3.7×10^5^ cells per 6-cm dish. After 24 h the cells were transiently transfected with 4.8 µg of C/EBPβ in pcDNA3.1+ expression vector using Lipofectamine 2000 transfection reagent (Invitrogen) and incubated for 24 h. NHK were seeded into 6-well plates as described above, transiently transfected with pGL3-CCL20 and stimulated with PMA for 24 h. Supernatants from transfected NHK and RTS3b as well as retrovirally infected NHK and HaCaT were collected and CCL20 protein levels were quantified using the ELISA duo-set kit for human CCL20 (R&D Systems).

### Quantitative real-time PCR

RNA was isolated using RNeasy Mini Kit (Qiagen, Hilden, Germany) and cDNA was generated from 1 µg of RNA with the First Strand cDNA Synthesis kit (Fermentas, St. Leon-Rot, Germany). Real-time PCR was performed with the LightCycler 1.5 instrument (Roche). PCR primers (Operon, Cologne, Germany) and probes (Roche Universal Probe Library; Roche) were designed using the Probe Finder software version 2.35 (Roche). The 66-bp fragment of CCL20 was detected with 5′-GCAGTCAAAGTTGCTTGCTTC-3′ and 5′-GCTGCTTTGATGTCAGTGC-3′ primer pair and probe no. 39. Glyceraldehyde 3-phosphate dehydrogenase (GAPDH) was detected with 5′-CTGTAGCCAAATTCGTTGT-3′and 5′-CTGACTTCAACAGCGACACC-3′ primer pair and probe no. 25, β-actin was detected with 5′-CCAACCGCGAGAAGATGA-3 and 5′-CCAGAGGCGTACAGGGATAG-3′ and probe no. 64. Changes in CCL20 expression were quantified using Roche Biochemicals LightCycler software data analysis version 3.5.28 (Roche).

### Electromobility shift assays

Putative binding sites for C/EBP were identified using the MatInspector program from Genomatix (Release 7.3.1) [35]. Oligonucleotides (Eurogentech, Brussels, Belgium) comprising these binding sites were labeled with (γ-^32^P) ATP using T4 polynucleotide kinase (New England Biolabs, Ipswich, MA). Nuclear extracts from retrovirally infected HaCaT cells or extracts containing GST-fusion proteins were mixed with the EMSA buffer (10 mM Tris-HCl (pH 7.5), 50 mM NaCl, 1 mM EDTA, 15% glycerol, 1 mg/ml of bovine serum albumin, 25 µg/ml poly(dI-dC), 11.5 mM DTT, 26 µg/ml aprotinin, 1.5 mM phenylmethylsulphonyl fluoride and 0.5 mg/ml salmon sperm DNA) and incubated at room temperature for 20 minutes. DNA-bound complexes were analyzed on a non-denaturing 4% polyacrylamide gel and visualized by autoradiography. Quantity One analysis software was used for quantification.

### Chromatin immunoprecipitation (ChIP) assay

RTS3b cells were seeded at 1×10^6^ cells per 10-cm dish and transiently transfected with 13.2 µg of C/EBPβ expression vector using FuGene 6 transfection reagent according to the manufacturer's guideline (Roche). After 24 h transfected RTS3b or retrovirally infected HaCaT cells were used for ChIP assay according to the protocol of Upstate Biotechnology (Lake Placid, NY) as described previously [72]. The cleared supernatant was incubated overnight at 4°C with 2 µg of C/EBPβ monoclonal H-7 mouse antibody (sc-7962, Santa Cruz Biotechnology) or mouse IgG2a kappa (M-7769, Sigma Aldrich Chemicals) per mg protein. The immune complexes were precipitated with 25 µl protein G Sepharose (Santa Cruz Biotechnology) in the presence of salmon sperm DNA for 1 h at 4°C. After elution of the protein-DNA complexes and reversing the cross-links, DNA was isolated and the CCL20 promoter region was detected and quantified by real-time PCR as described above using the 5′-AGCAAATATTGGGAATGTACACAG-3′ and 5′-CTTCGCACCTTCCCAATATG-3′ primer pair together with probe no. 4. Finally, PCRs were repeated from the same precipitated DNA and visualized on 2.5% agarose gels.

### Statistical analysis

To evaluate the statistical differences between analyzed groups, a two-sided unpaired t-test was applied.

### Databases

In the Swissprot database (http://www.uniprot.org) CCL20 is available under the accession number P78556 and C/EBPβ under the accession number P17676. The following accession numbers of the GenBank database (http://www.ncbi.nlm.nih.gov/nuccore/) were used to obtain sequence records: AY150053 for the CCL20 promoter sequence and NM_001130046.1 for the CCL20 mRNA, NM_005194 for the mRNA of C/EBPβ and M12737.1 for the HPV8 genomic sequence.

## Supporting Information

Figure S1 **Reduction of Langerhans cells and CCL20 in EV lesions compared with normal human epidermis.** The numbers of Langerhans cells were counted in 8 different areas of normal human epidermis and HPV8-positive EV lesions, respectively (A). Sections of normal human epidermis (B, C) or HPV8-positive EV lesions (D) were stained using an antibody against CCL20 and hematoxylin in the absence (B, D) or presence (C) of CCL20 protein as a blocking reagent. Bars correspond to 20 µm in B–D.
(TIF)

Figure S2 **C/EBPβ and CCL20 staining in serial sections of normal human epidermis.** Consecutive serial sections of normal human epidermis were stained with antibodies directed against C/EBPβ (left panel) and CCL20 (right panel) and hematoxylin. Bars correspond to 20 µm. Nuclei strongly positive for C/EBPβ and CCL20 expressing cells in the consecutive section are marked with arrows in the respective panels.
(TIF)

Figure S3 **Mutated CCL20 promoter-proximal C/EBP binding sites do not bind the C/EBP transcription factors.** ^32^P-labeled oligonucleotides containing the wild-type or mutated C/EBP binding sites (nt 716–724, nt 734–748) of the CCL20 promoter were incubated with GST, GST-C/EBPα or GST-C/EBPβ fusion proteins and analyzed by EMSA. The arrow indicates complexes corresponding to C/EBP DNA binding activity.
(TIF)

Figure S4 **TNF-α- or poly(I∶C)-induced CCL20 promoter activation depends on NF-κB but not C/EBP binding sites.** NHK were transfected with luciferase reporter constructs either under the control of the wild-type CCL20 promoter (black bars), the CCL20 promoter containing mutations of the proximal NF-κB binding site (white bars) or mutations in the two promoter-proximal C/EBP binding sites (grey bars). 15 h post-transfection the cells were stimulated with TNF-α (1000 U/ml, Bender&Co., Vienna, Austria), poly(I∶C) (1 µg/ml, Novagen, Darmstadt, Germany) or medium as a control. 24 h later luciferase activity was determined and normalized to protein concentration of the respective luciferase extract. The normalized luciferase activities of the control transfections for each reporter construct were set at 1. Transfections were conducted in triplicates.
(TIF)

Figure S5 **HPV8 E7 binds to C/EBPβ and suppresses its activity via its carboxy-terminus.** (A) RTS3B cells were transfected with CCL20 promoter luciferase construct (0.5 µg) and C/EBPβ (0.2 µg) in the presence or absence of Flag-tagged HPV8 E7 (0.5 µg) or its deletion mutants. Total amount of DNA was adjusted with pCMV-Flag2 empty vector. After 24 h the luciferase activity was measured and normalized to protein concentration of the respective luciferase extract. The normalized luciferase activity of the control transfection was set at 1. Transfections were conducted in triplicates. Shown are mean values from two independent experiments ± SD. Asterisks represent statistical significance, ***p≤0.0005 (Flag-HPV8 E7 full length, E7Δ1-15, E7Δ1-37), **p = 0.0072 (Flag-HPV8 E7Δ86-90). Ns, not significant (Flag-HPV8 E7Δ79-83). (B) Identical extracts were analyzed by Western blot (WB) with anti-Flag antibodies (upper panel) and anti-actin as loading control for the whole cell extracts (lower panel).
(TIF)

Figure S6 **HPV8 E7 suppresses CCL20 expression in HaCaT cells.** Stable mRNA expression of HPV8 E7 (pLXSN-HPV8 E7) after retroviral gene transfer in HaCaT cells (A) and HPV8 E6 (pLXSN-HPV8 E6) and E7 (pLXSN-HPV8 E7) oncogenes in NHK (B) was verified by quantitative PCR. After RNA isolation and cDNA synthesis, the 89-bp fragment of E6 was amplified by PCR with 5′-ccgcaacgtttgaatttaatg-3′ and 5′-attgaacgtcctgtagctaattca-3′ primer pair and the 76-bp fragment of E7 with 5′-aggaattaccaaacgaacagga-3′ and 5′-cacggtgcaacaattttgaata-3′ primer pair and visualized on agarose gels. CCL20 mRNA (C) and protein (D) levels were quantified in HaCaT cells stably expressing the E7 oncogene and respective control cells. The amount of CCL20 mRNA (in relation to β-actin as measured by quantitative real-time PCR) or protein in control cells was set at 1. CCL20 protein levels in supernatants were determined by ELISA. Measurements represent the mean values ± SD from two independent retroviral infections. Asterisks represent statistical significances, p<0.0001.
(TIF)
