# Supplementary figures and images for: Human Papillomavirus Type 8 Interferes with a Novel C/EBPβ-Mediated Mechanism of Keratinocyte CCL20 Chemokine Expression and Langerhans Cell Migration
Source: PLoS Pathog. 2012 Jul 26;8(7):e1002833. doi: 10.1371/journal.ppat.1002833 (PMC3406103; doi:10.1371/journal.ppat.1002833)

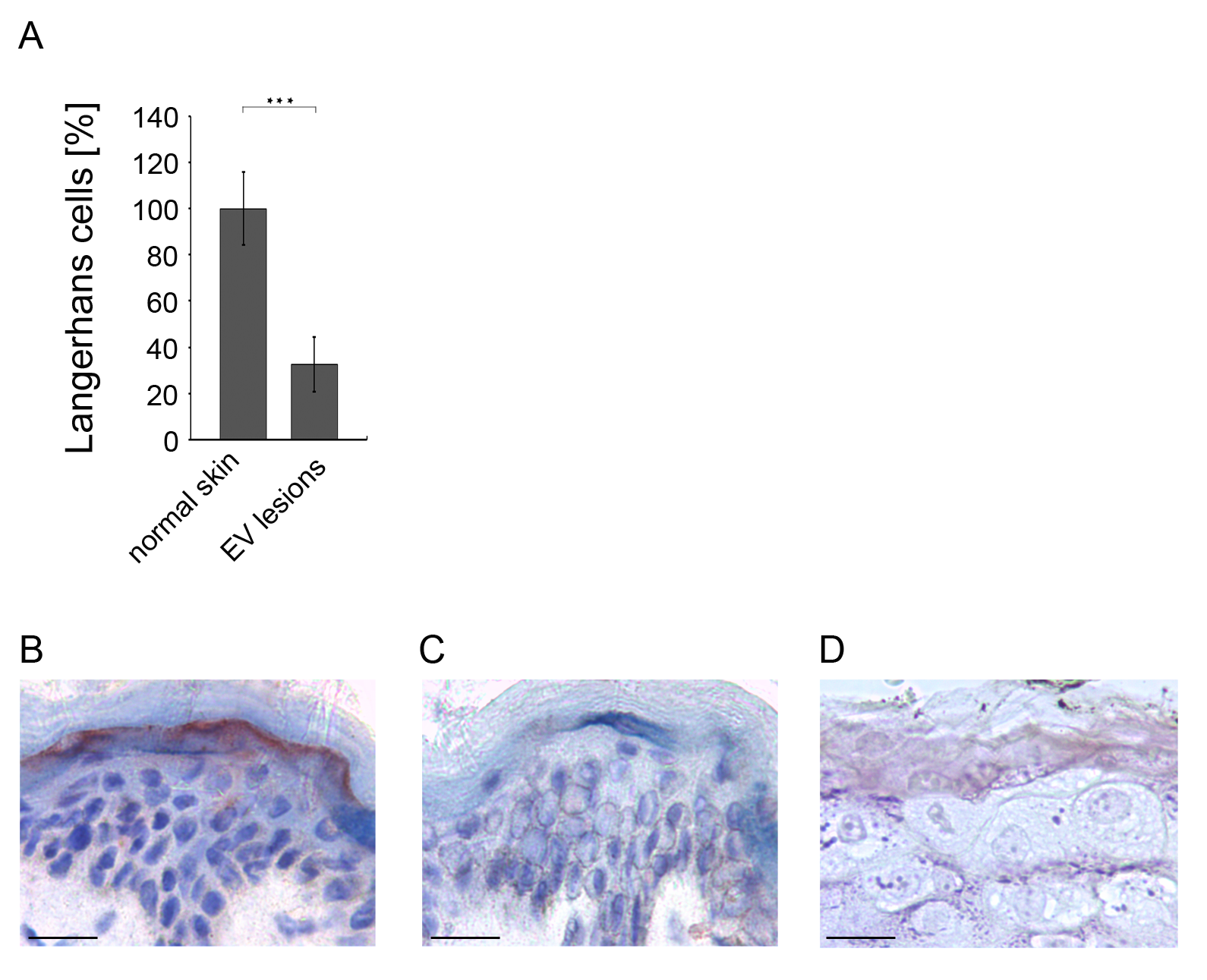

Supplement: Figure S1 — Reduction of Langerhans cells and CCL20 in EV lesions compared with normal human epidermis. The numbers of Langerhans cells were counted in 8 different areas of normal human epidermis and HPV8-positive EV lesions, respectively (A). Sections of normal human epidermis (B, C) or HPV8-positive EV lesions (D) were stained using an antibody against CCL20 and hematoxylin in the absence (B, D) or presence (C) of CCL20 protein as a blocking reagent. Bars correspond to 20 µm in B–D. (TIF) [file ppat.1002833.s001.tif]

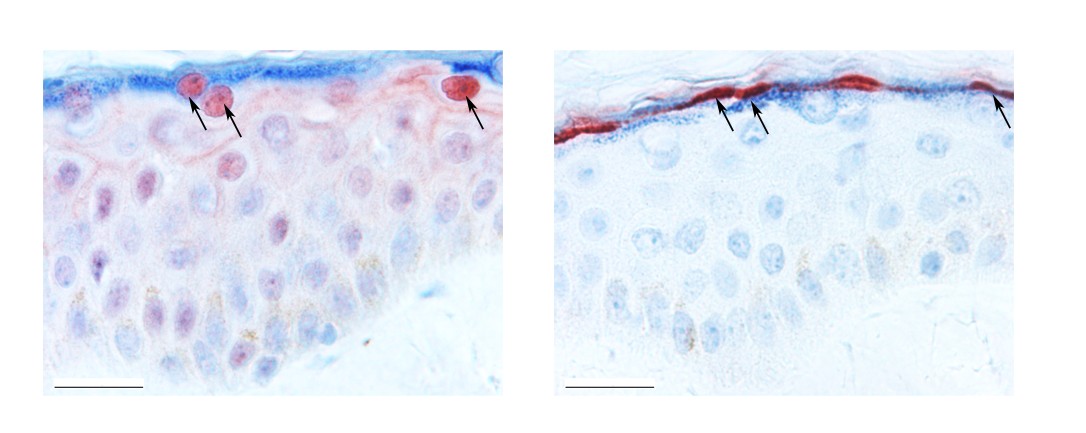

Supplement: Figure S2 — C/EBPβ and CCL20 staining in serial sections of normal human epidermis. Consecutive serial sections of normal human epidermis were stained with antibodies directed against C/EBPβ (left panel) and CCL20 (right panel) and hematoxylin. Bars correspond to 20 µm. Nuclei strongly positive for C/EBPβ and CCL20 expressing cells in the consecutive section are marked with arrows in the respective panels. (TIF) [file ppat.1002833.s002.tif]

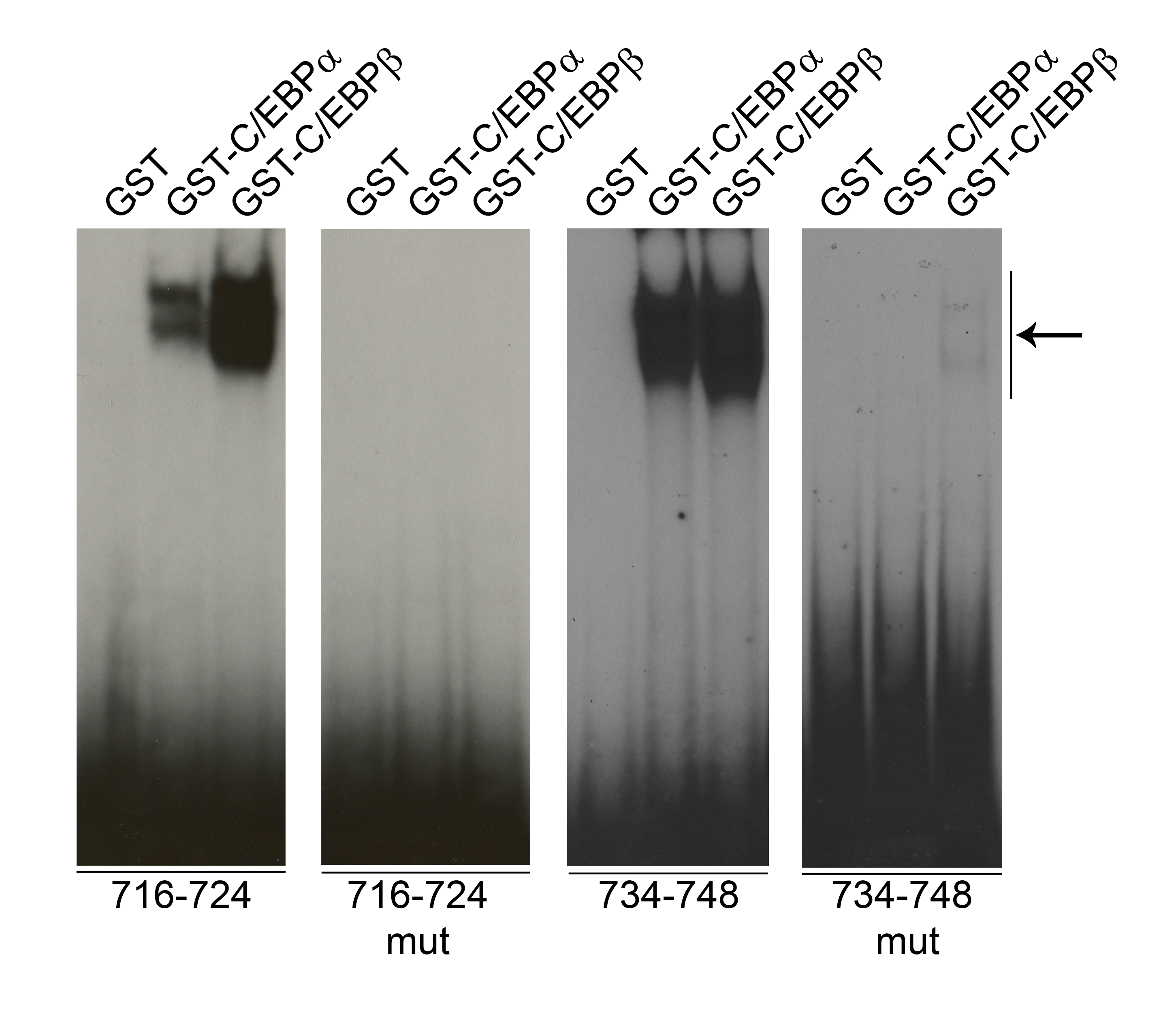

Supplement: Figure S3 — Mutated CCL20 promoter-proximal C/EBP binding sites do not bind the C/EBP transcription factors. 32P-labeled oligonucleotides containing the wild-type or mutated C/EBP binding sites (nt 716–724, nt 734–748) of the CCL20 promoter were incubated with GST, GST-C/EBPα or GST-C/EBPβ fusion proteins and analyzed by EMSA. The arrow indicates complexes corresponding to C/EBP DNA binding activity. (TIF) [file ppat.1002833.s003.tif]

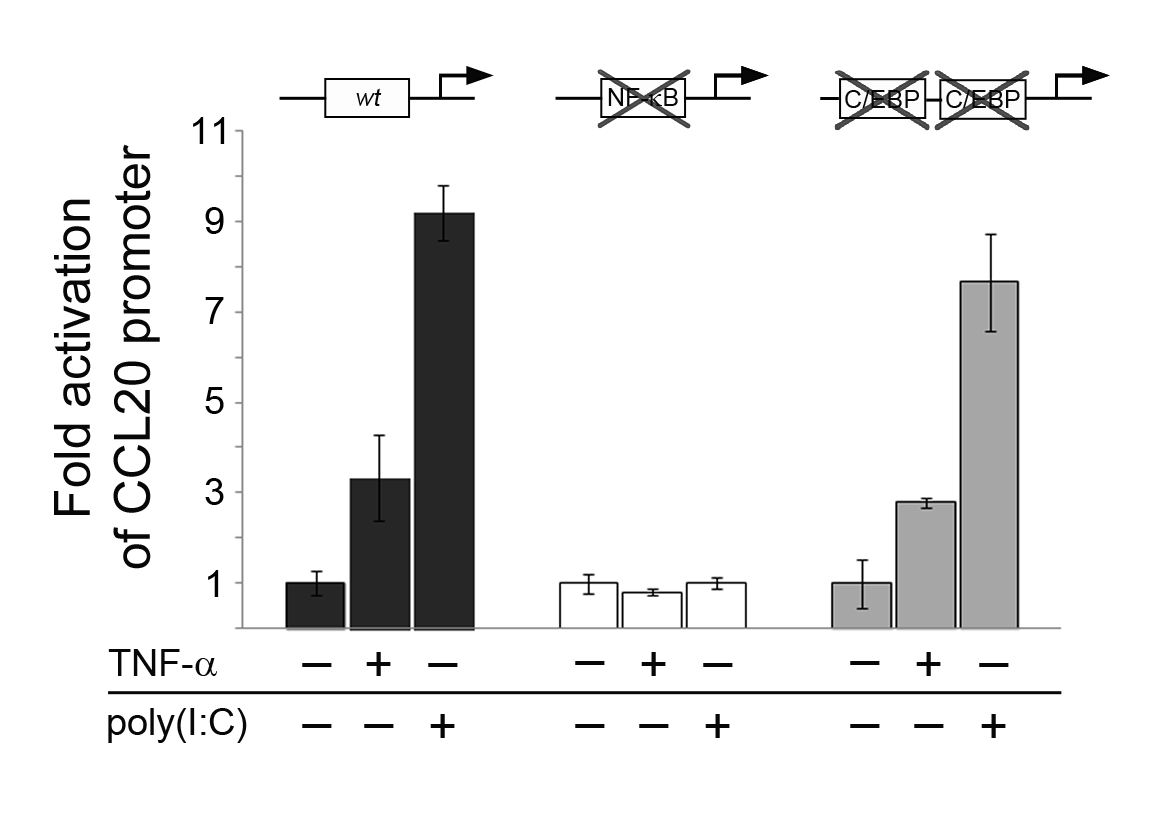

Supplement: Figure S4 — TNF-α- or poly(I∶C)-induced CCL20 promoter activation depends on NF-κB but not C/EBP binding sites. NHK were transfected with luciferase reporter constructs either under the control of the wild-type CCL20 promoter (black bars), the CCL20 promoter containing mutations of the proximal NF-κB binding site (white bars) or mutations in the two promoter-proximal C/EBP binding sites (grey bars). 15 h post-transfection the cells were stimulated with TNF-α (1000 U/ml, Bender&Co., Vienna, Austria), poly(I∶C) (1 µg/ml, Novagen, Darmstadt, Germany) or medium as a control. 24 h later luciferase activity was determined and normalized to protein concentration of the respective luciferase extract. The normalized luciferase activities of the control transfections for each reporter construct were set at 1. Transfections were conducted in triplicates. (TIF) [file ppat.1002833.s004.tif]

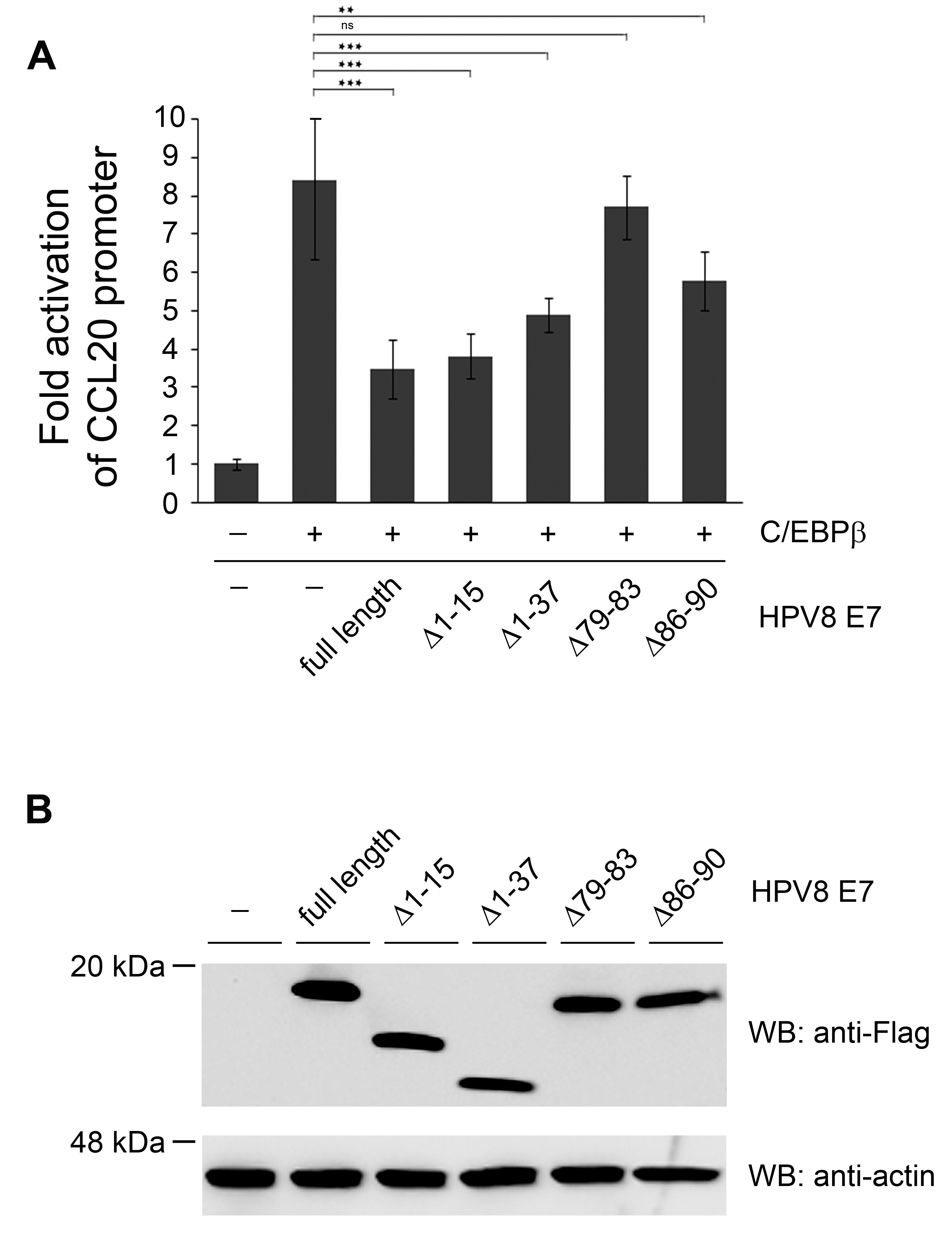

Supplement: Figure S5 — HPV8 E7 binds to C/EBPβ and suppresses its activity via its carboxy-terminus. (A) RTS3B cells were transfected with CCL20 promoter luciferase construct (0.5 µg) and C/EBPβ (0.2 µg) in the presence or absence of Flag-tagged HPV8 E7 (0.5 µg) or its deletion mutants. Total amount of DNA was adjusted with pCMV-Flag2 empty vector. After 24 h the luciferase activity was measured and normalized to protein concentration of the respective luciferase extract. The normalized luciferase activity of the control transfection was set at 1. Transfections were conducted in triplicates. Shown are mean values from two independent experiments ± SD. Asterisks represent statistical significance, ***p≤0.0005 (Flag-HPV8 E7 full length, E7Δ1-15, E7Δ1-37), **p = 0.0072 (Flag-HPV8 E7Δ86-90). Ns, not significant (Flag-HPV8 E7Δ79-83). (B) Identical extracts were analyzed by Western blot (WB) with anti-Flag antibodies (upper panel) and anti-actin as loading control for the whole cell extracts (lower panel). (TIF) [file ppat.1002833.s005.tif]

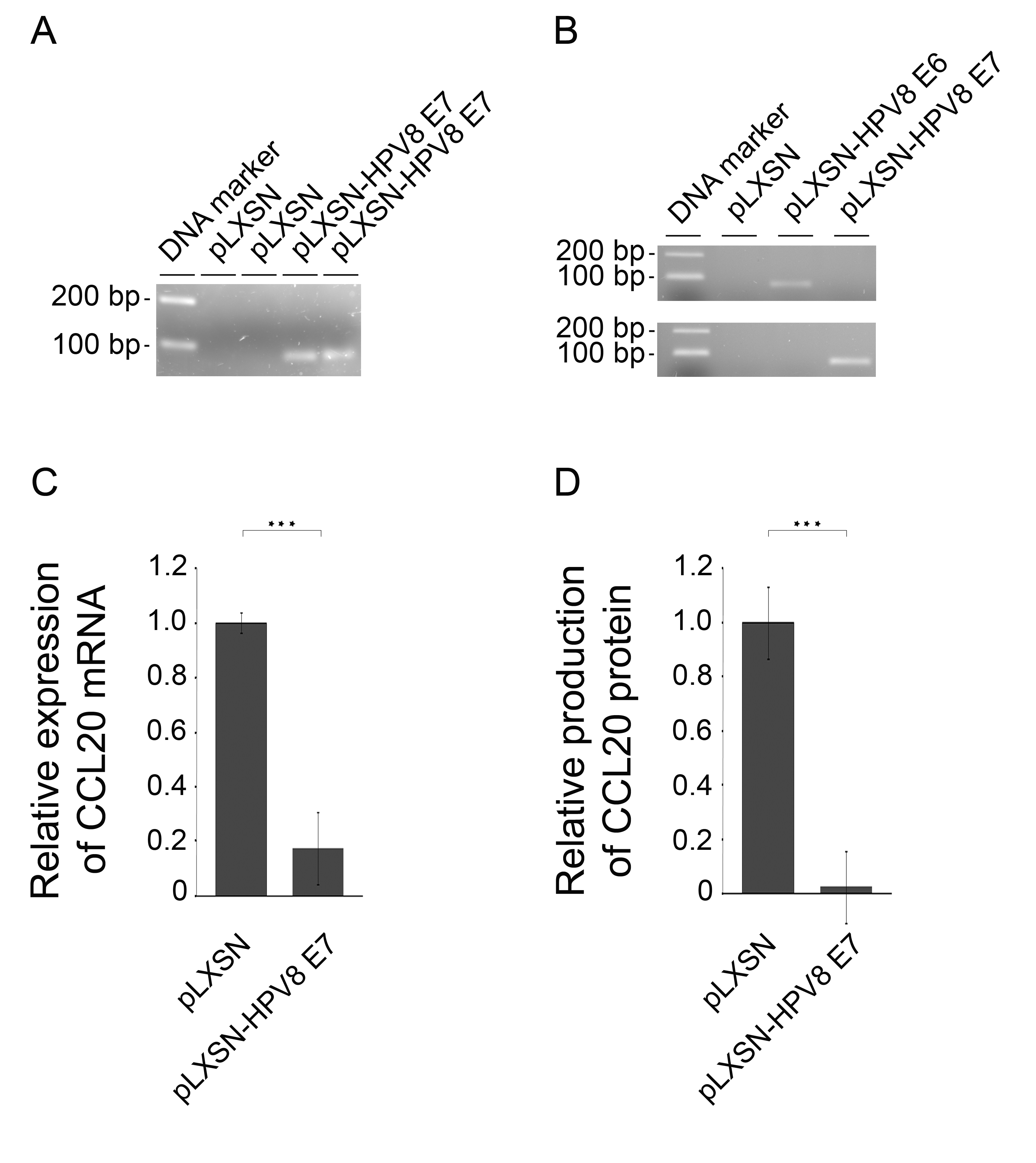

Supplement: Figure S6 — HPV8 E7 suppresses CCL20 expression in HaCaT cells. Stable mRNA expression of HPV8 E7 (pLXSN-HPV8 E7) after retroviral gene transfer in HaCaT cells (A) and HPV8 E6 (pLXSN-HPV8 E6) and E7 (pLXSN-HPV8 E7) oncogenes in NHK (B) was verified by quantitative PCR. After RNA isolation and cDNA synthesis, the 89-bp fragment of E6 was amplified by PCR with 5′-ccgcaacgtttgaatttaatg-3′ and 5′-attgaacgtcctgtagctaattca-3′ primer pair and the 76-bp fragment of E7 with 5′-aggaattaccaaacgaacagga-3′ and 5′-cacggtgcaacaattttgaata-3′ primer pair and visualized on agarose gels. CCL20 mRNA (C) and protein (D) levels were quantified in HaCaT cells stably expressing the E7 oncogene and respective control cells. The amount of CCL20 mRNA (in relation to β-actin as measured by quantitative real-time PCR) or protein in control cells was set at 1. CCL20 protein levels in supernatants were determined by ELISA. Measurements represent the mean values ± SD from two independent retroviral infections. Asterisks represent statistical significances, p<0.0001. (TIF) [file ppat.1002833.s006.tif]
